# Realizing Mitigation Efficiency of European Commercial Forests by Climate Smart Forestry

**DOI:** 10.1038/s41598-017-18778-w

**Published:** 2018-01-10

**Authors:** Rasoul Yousefpour, Andrey Lessa Derci Augustynczik, Christopher P. O. Reyer, Petra Lasch-Born, Felicitas Suckow, Marc Hanewinkel

**Affiliations:** 1 Chair of Forestry Economics and Forest Planning, Faculty of Environment and Natural Resources, University of Freiburg, Tennenbacherstr. 4, D-79106 Freiburg, Germany; 2 Potsdam Institute for Climate Impact Research (PIK), Telegraphenberg A62/1.05, D-14412 Potsdam, Germany

## Abstract

European temperate and boreal forests sequester up to 12% of Europe’s annual carbon emissions. Forest carbon density can be manipulated through management to maximize its climate mitigation potential, and fast-growing tree species may contribute the most to Climate Smart Forestry (CSF) compared to slow-growing hardwoods. This type of CSF takes into account not only forest resource potentials in sequestering carbon, but also the economic impact of regional forest products and discounts both variables over time. We used the process-based forest model 4 C to simulate European commercial forests’ growth conditions and coupled it with an optimization algorithm to simulate the implementation of CSF for 18 European countries encompassing 68.3 million ha of forest (42.4% of total EU-28 forest area). We found a European CSF policy that could sequester 7.3–11.1 billion tons of carbon, projected to be worth 103 to 141 billion euros in the 21st century. An efficient CSF policy would allocate carbon sequestration to European countries with a lower wood price, lower labor costs, high harvest costs, or a mixture thereof to increase its economic efficiency. This policy prioritized the allocation of mitigation efforts to northern, eastern and central European countries and favored fast growing conifers *Picea abies* and *Pinus sylvestris* to broadleaves *Fagus sylvatica* and *Quercus* species.

## Introduction

Human population growth and increasing demand for energy and other resources have led to a strong intensification of GHG emissions during the past century^1^, driving global climate change^2,3^. Considering the threats imposed by climate change on ecosystems and the corresponding direct impact on human well-being, mitigating human-induced climate change is essential^4^. Here, forests play a central role in climatic stabilization and sequestering carbon in biomass and soil^5^. Currently, the role of forests and forest management for carbon sequestration as a mitigation strategy is an area of active debate^6–8^. Climate Smart Forestry, i.e. forest management that recognizes synergies among climate change mitigation and other forest benefits and optimizes the effectiveness (magnitude) and efficiency (cost) of forests’ contributions to climate change mitigation, is seen as a way to contribute to the ambitious COP 21 goals at a European level^9,10^. Therefore, we focused our study on increasing forest carbon sequestration as foreseen by the Kyoto protocol and applied an econometric approach for optimizing the mitigation solutions.

Despite the availability of literature concerning adaptive management strategies^11,12^ and economic evaluations of climate change impacts^13^, a detailed species- and country-specific economic evaluation of forest mitigation policies that we consider to be “Climate Smart Forestry” at a large scale, i.e. the European level, is still missing^14^. In this context, we defined the main objectives of this study as (i) Assessing the economic implications of realizing CSF in Europe regarding different forestry-relevant species, (ii) Identifying target areas for adopting efficient mitigation, and (iii) Analyzing sensitivity of CSF outcomes (supply-cost frontiers) to economic parameters (e.g. discount rate). We also took into account future climate conditions affecting our main objectives (i–iii).

Forest carbon sequestration and density can be manipulated through controlling forest utilization, i.e. harvesting rates and forest stands’ rotation length^9^. Aiming for an efficient balance of carbon sequestration and forest profitability, we considered seven policy alternatives (A-G) presenting the preferences for carbon sequestration and NPV (see Table 1). Each Policy could be implemented using an optimized combination of management strategies depending on the forests types, climate scenarios, and economic conditions.

**Table 1:** Weight combinations for the policies.

Policy Scheme	Name	Weight for NPV	Weight for Carbon sequestration
A	Max NPV	1	0
B	Balanced a	0.67	0.33
C	Balanced b	0.57	0.43
D	Balanced c	0.5	0.5
E	Balanced d	0.43	0.57
F	Balanced e	0.33	0.67
G	Max carbon sequestration	0	1

**Figure 1:**
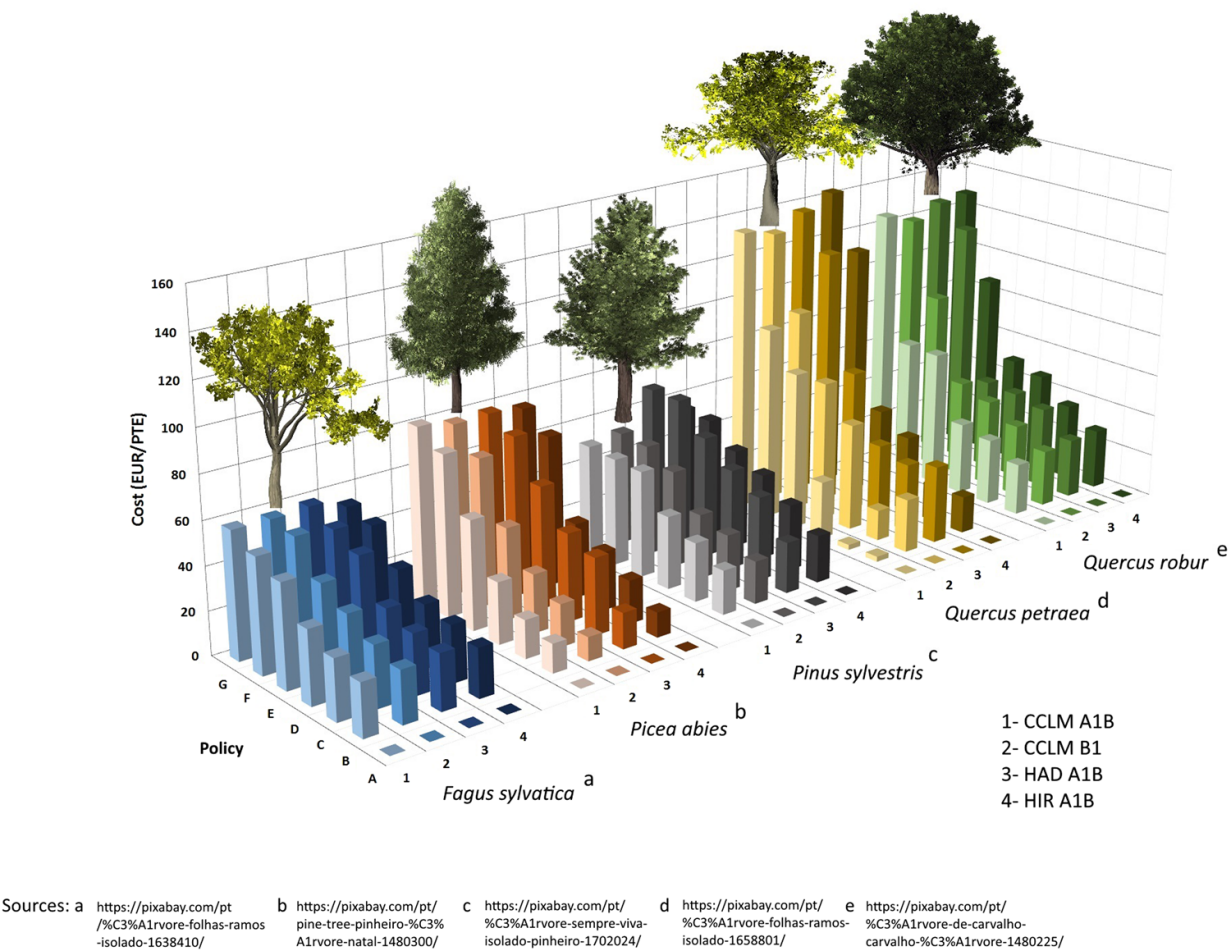
Costs for an efficient carbon forestry in European countries and major tree species. The figure shows the development of opportunity costs for different policies with increasing preference for carbon sequestration from A to G, for all countries, species, and four climate change trajectories. Figures are discounted by country specific rates to calculate the monetary NPV (Net Present Value) of cash flows and 2% for carbon sequestration to get the current sequestration potential in PTE (Present Ton Equivalent). The tree images were retrieved from Pixabay database (^1^ https://pixabay.com/pt/%C3%A1rvore-folhas-ramos-isolado-1638410/; ^2^ https://pixabay.com/pt/pine-tree-pinheiro-%C3%A1rvore-natal-1480300/; ^3^ https://pixabay.com/pt/%C3%A1rvore-sempre-viva-isolado-pinheiro-1702024/; ^4^ https://pixabay.com/pt/%C3%A1rvore-folhas-ramos-isolado-1658801/; ^5^ https://pixabay.com/pt/%C3%A1rvore-de-carvalho-%C3%A1rvore-1480225/).

The table presents the preferences for carbon sequestration and NPV for the policies depicted in Fig. 1.

The management strategies were composed of four types of interventions: 1- Forest conservation (no management); 2- Business as usual (BAU); 3- Intensified forest wood harvest and 4- Decreased forest wood harvest (details in Supplementary 2). When selecting appropriate management strategies, it is crucial to consider changes in forest productivity under new climatic conditions and increasing concentration of CO_2_ in the atmosphere^15^. Therefore, we considered four trajectories of future climate change (CCLM A1B/B1, HIR A1B, and HAD A1B) representing the medium range of global warming expected under current mitigation policy pledges^16^ and two CO_2_ concentration pathways (see details in Supplementary 1). CCLM B1 and HIR A1B both represented the climate target of max. 2 °C global surface warming at the end of 21^st^ century, and CCLM A1b and HAD A1B model a higher target, i.e. above 4 °C warming (see Fig. 4 in Supplementary 1). We applied the process-based forest growth model 4 C (see Methods for details) for analyzing European commercial forests’ responses in terms of wood production and carbon sequestration for the five most abundant commercial species, in eighteen countries, and four climate change trajectories. Forest growth model 4 C simulates forest responses and changes in forest structure, leaf area index, carbon and water balance to climatic conditions and various management interventions, including harvesting and thinning (details in Methods).

To optimize Climate Smart Forestry for European forests, we applied a coupled ecological-economic framework incorporating economic factors along with ecological potentials. We used a multi-objective optimization approach to compute the trade-off between carbon sequestration and commercial wood production using 4 C simulation outputs. For discounting future cash flows, we applied the country-specific long-term interest rate defined by the European Central Bank and referred to the figures as net present value (NPV). A carbon discounting factor of 2% was applied to calculate present tons equivalent of carbon (PTE). Both discounting schemes were implemented to simultaneously include the effects of economic and carbon fluctuations over time and time preferences about when the income and sequestration occurs^17^. Moreover, a 0% discount rate was applied for the scenario where there is no urgency for realizing CSF targets. For all these cases, we found the optimal management alternatives for equal (no preference) and multiple varying weight combinations of discounted NPV and carbon sequestration (PTE) through a goal programming optimization model (see Table 1). We accounted for carbon cost as the opportunity costs of favoring additional carbon sequestration compared to the economically optimal forest utilization with the maximum NPV. We expected that with higher forest profitability, i.e. higher wood prices and low harvesting costs, higher opportunity costs for sequestering carbon would occur. Similarly, with lower interest rates, forest profitability would increase and this would lead to higher costs. Accordingly, areas with low carbon costs and high sequestration potential were identified as target areas for efficient mitigation policies.

## Results

### Carbon cost in Europe

The carbon costs for the species included in our study are shown in Fig. 1. The costs were weighted by the sequestration potential in each country, for different policies A to G with increasing preference for carbon sequestration over NPV as described in Table 1. Our results indicated the highest costs occurring for *Quercus* species, especially when the preference for carbon sequestration is high (Policy E-G). Considering the implementation of the maximum sequestration potential (policy G), the costs ranged from 129.89 to 143.49 EUR/PTE for *Quercus petraea* and from 120.27 to 128.03 EUR/PTE for *Quercus robur*. *Fagus sylvatica* presented the lowest cost range, with the European average ranging from 53.11 to 57.40 EUR/PTE as a result of the low wood price for this species. Moderate carbon sequestration opportunity costs were found for conifers with high carbon sequestration potentials, e.g. *Picea abies* and *Pinus sylvestris* with 76.98 to 81.39 EUR/PTE and 50.77 to 96.52 EUR/PTE respectively. The opportunity costs for sequestering carbon were decreased with the increased preference for NPV, as displayed in Fig. 1. With equal preference for carbon and NPV (policy D), the costs decreased by 54% on average, compared to policy G. Overall, the maximum carbon costs for Europe were estimated to range from 50.77 to 143.49 EUR/PTE depending on climate change trajectory and species considered.

**Figure 2:**
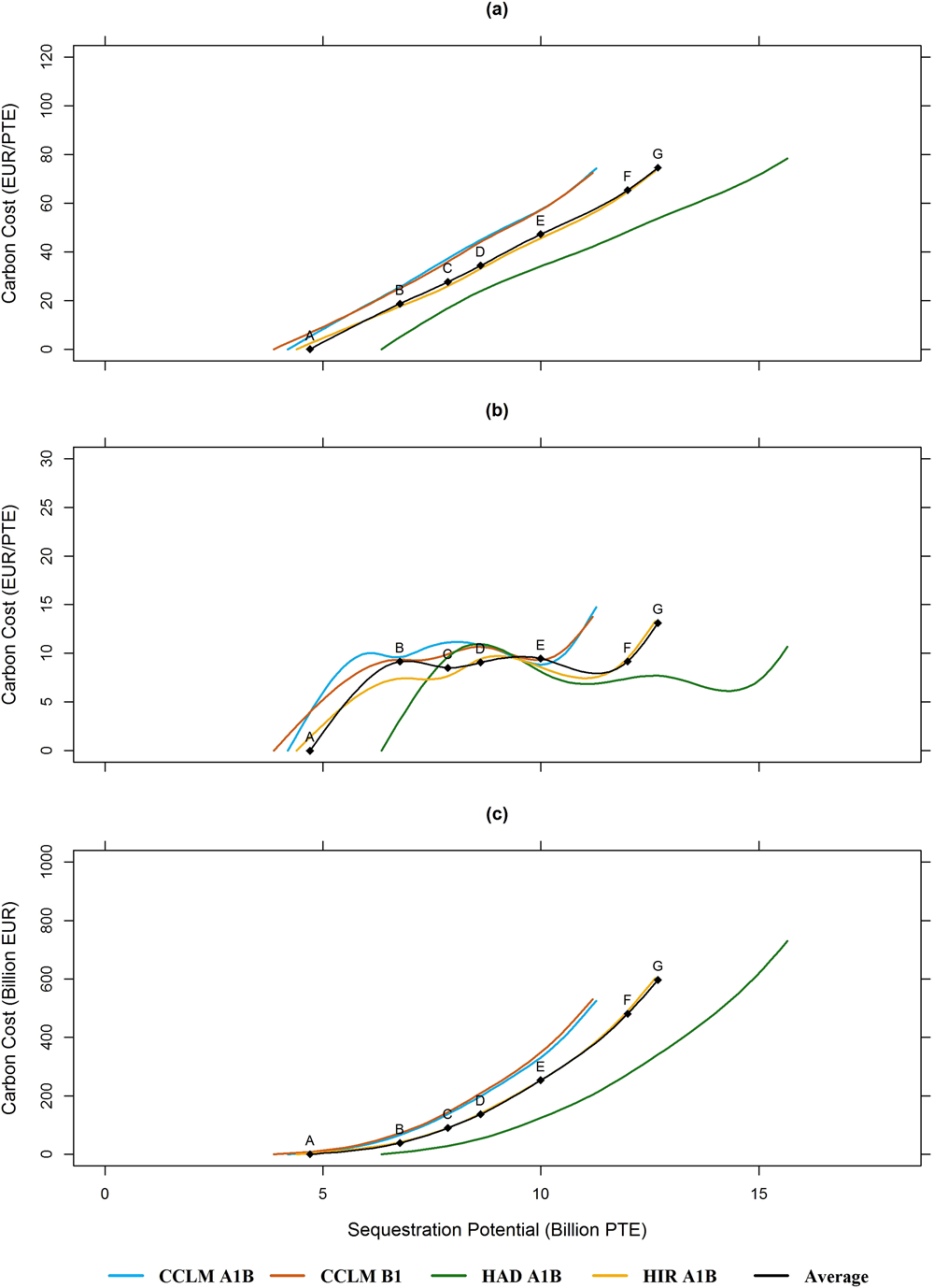
Carbon supply, marginal and total cost regarding different future climate change trajectories. The figure shows the increase on carbon costs in Europe required for increasing the sequestration potential in 1 Billion PTE (discounted carbon with 2% as Present Ton Equivalent) at different sequestration levels, for each climate change scenario considered.

The European carbon sequestration supply curves are displayed in Fig. 2, considering the countries and species included in our analysis (details in Supplementary 1) and representing an area of 68.3 million hectares (42.4% of the total forest area in EU-28). The carbon sequestration supply curves in Europe for each climate change trajectory are shown in Fig. 2a, with the average response of the four climate change trajectories indicated in black. The marginal costs are displayed in Fig. 2b, i.e. the acceleration of increase in carbon cost with increasing (policy) preference for carbon sequestration, and the total cost at each carbon sequestration level is shown in Fig. 2c.

The maximum carbon sequestration potential of European forests (policy G) was estimated to range from 11.18 to 15.65 billion PTE using the 4 C model for the period 2010–2090 (12.7 billion PTE on average, Fig. 2a). However, realizing this potential would incur a total carbon cost of 596.9 billion EUR (Fig. 2c), i.e. 74.59 EUR/PTE, which equals two thirds of the maximum economic profit from commercial wood utilization in European forests (906.2 billion EUR). This is moderately higher than average global carbon cost estimates, which are about 53 EUR/tC^18^. With increasing preference for NPV, sequestration costs decreased, resulting in lower sequestration. For example, with equal preference for carbon sequestration and NPV (point D), a sequestration potential of 8.6 billion PTE was achieved with sequestration costs of 34.35 EUR/PTE.

The cost curves were shifted to the right under HIR A1B and HAD A1B trajectories (Fig. 2a), simulating a higher carbon sequestration potential (>15 billion PTE) compared to both CCLM trajectories (<12 billion PTE) at an equal carbon cost. The cost of sequestering an additional unit (e.g. PTE) of carbon in forests, i.e. the marginal cost of carbon, resulted in carbon cost curves with a negative skewness (Fig. 2b) and the greatest marginal costs are expected at the early and final stages of implementing CFS.

**Figure 3:**
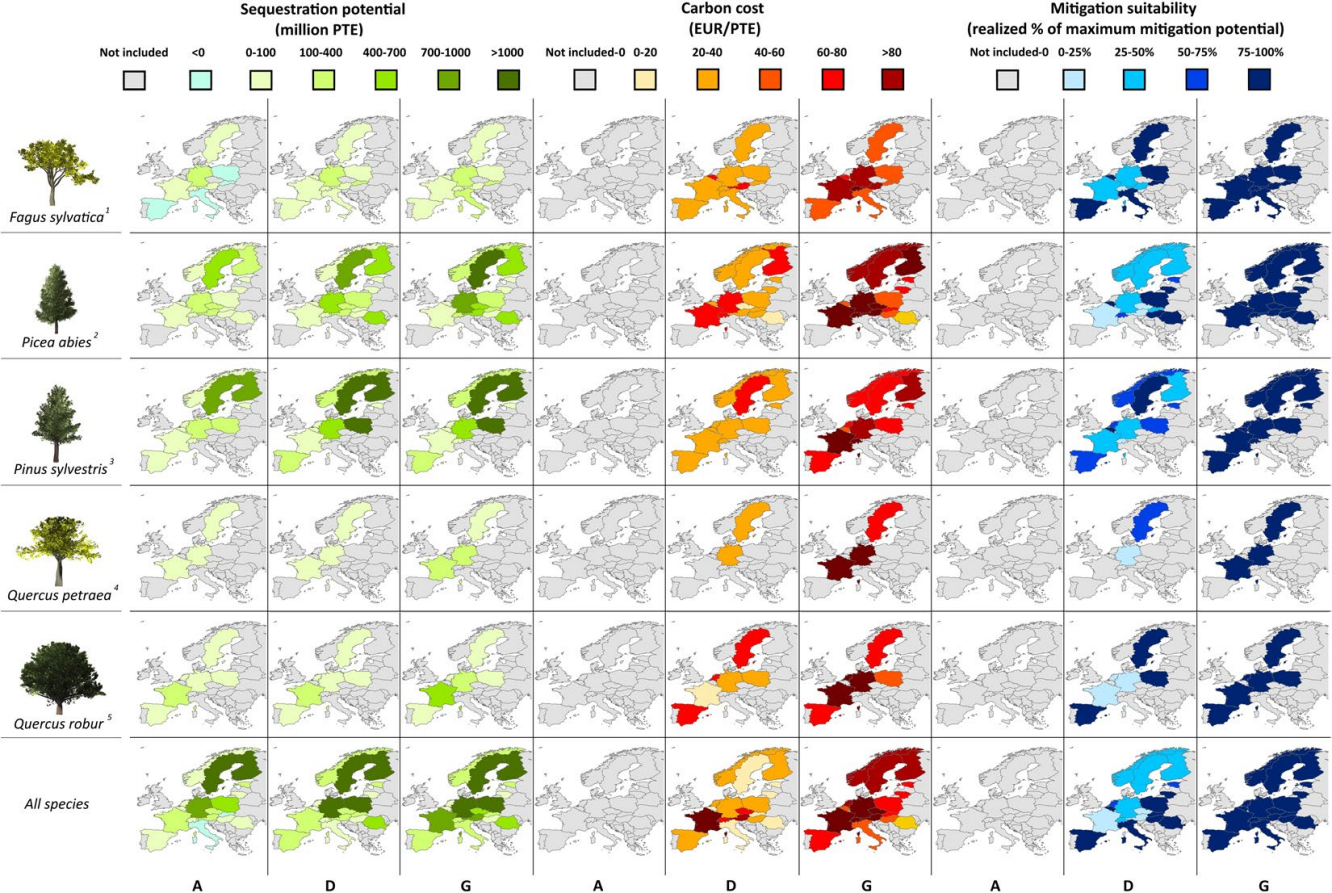
Carbon sequestration potential, carbon cost, and CSF allocation to European economically low costs regions. Policies A, D, and G aim for realizing maximum NPV, CSF, and maximum Mitigation, respectively (see details in Table 1). Figure 3 was created using the software QGIS Version 2.18.13 (http://www.qgis.org/en/site/) and paint.net version 4.0.12 (https://www.getpaint.net/). The base map was made with Natural Earth. Free vector and raster map data @ naturalearthdata.com and the tree images were retrieved from Pixabay database (^1^ https://pixabay.com/pt/%C3%A1rvore-folhas-ramos-isolado-1638410/; ^2^ https://pixabay.com/pt/pine-tree-pinheiro-%C3%A1rvore-natal-1480300/; ^3^ https://pixabay.com/pt/%C3%A1rvore-sempre-viva-isolado-pinheiro-1702024/; ^4^ https://pixabay.com/pt/%C3%A1rvore-folhas-ramos-isolado-1658801/; ^5^ https://pixabay.com/pt/%C3%A1rvore-de-carvalho-%C3%A1rvore-1480225/).

The sequestration potential, carbon costs, and suitability of each species and country to achieve policy goals are shown in Fig. 3. The results represent an average of the four climate change trajectories for three distinct policies: A (max. NPV), D (CSF with equal preference for NPV and mitigation), and G (max. mitigation). *Picea abies* and *Pinus sylvestris* displayed the highest sequestration potential, because of higher growth rates and forest cover. At the country level, higher sequestration potential (>1000 million PTE) occurred in Finland, Sweden, Germany and Poland, as a result of the high forest coverage in the Scandinavian countries and high growth rates in Germany. Broadleaves (*Fagus sylvatica, Quercus* species) and southern Mediterranean countries (e.g. Italy, Spain) had the lowest sequestration potentials (<400 million PTE).

A high carbon cost was observed for *Quercus* species in Germany and France, surpassing 80 EUR/PTE (policy D). Moreover, France might face a high cost when considering *Pinus sylvestris* for CSF, and the same would apply to Germany and Finland for implementing a high carbon sequestration program with *Picea abies*. A comparable carbon cost (40–60 EUR/PTE) relative to the global estimated average cost (53 EUR/PTE) may be achieved for CSF under a moderate mitigation strategy with policy G.

A final step in implementing European CSF prioritized an early increase in carbon sequestration for species and countries with the lowest opportunity costs. The last three columns of Fig. 3 show the fraction of the maximum sequestration potential of the corresponding country and species to be exploited for the optimal implementation of CSF. We observed an early allocation of CSF to *Picea abies* in eastern European countries and *Fagus sylvatica* in Spain, Slovakia and Poland. Essentially, optimized CSF prioritized sequestration in these low-cost areas, whereas central Europe and Scandinavia remained as areas focusing on wood production. With increasing preference for carbon sequestration, low-cost areas were not capable of providing enough sequestration potential, demanding an increase in sequestration in Scandinavian countries and Germany (areas with high sequestration potential), while balancing carbon sequestration and forest profitability. Aiming for the maximum carbon sequestration (policy G), all areas were allocated to carbon sequestration. The most efficient species were *Fagus sylvatica* and *Pinus sylvestris*, were prioritized for carbon sequestration because of low costs for the former and the large forest cover with reasonable costs for the latter.

### Carbon Management in European Forests

**Figure 4:**
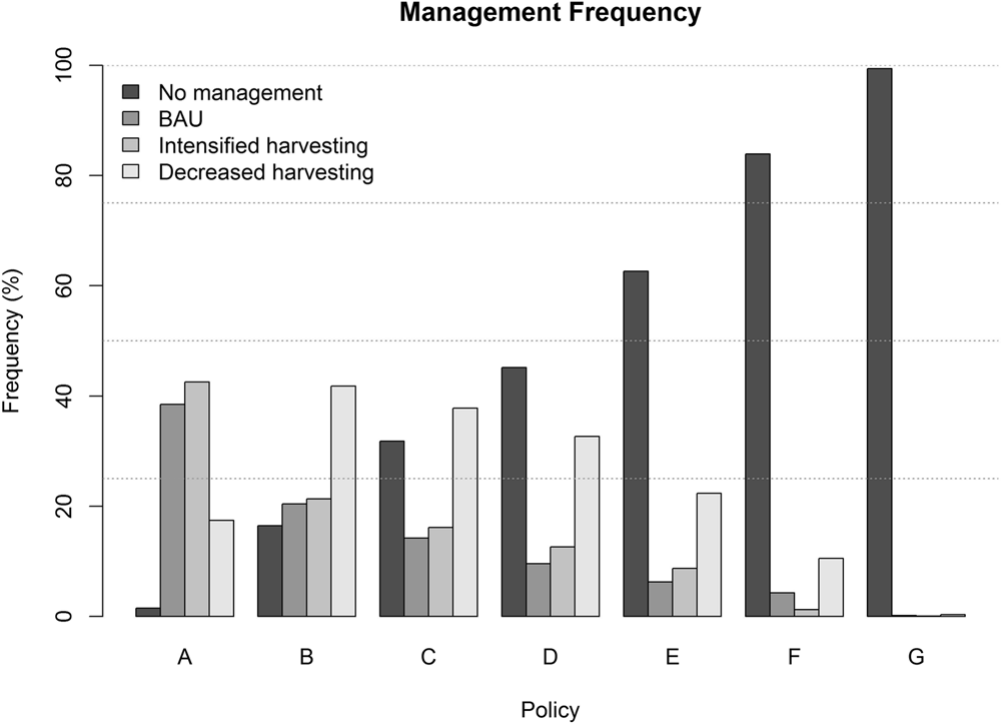
Frequency of forest management interventions to implement policies (A–G) in Europe.

According to Fig. 4, *in situ* carbon sequestration (i.e. non-management) was not the most efficient carbon sequestration strategy in our study for implementing a CSF with a moderate and efficient carbon sequestration (Policy D) causing some economic loss. In general, decreased wood utilization appeared as the main course of action to increase carbon sequestration and maintaining suitable profitability from wood harvest income. To realize optimal CSF, management strategies were mixed not only among species but also for a given species (details in Supplementary 2). BAU and intensified harvesting were both dominant strategies if economic concerns define forest management policy in Europe (policies A-D). In addition, the full spectrum of management strategies (specific forestry interventions, i.e. thinning) can be implemented if policies consider both NPV and carbon sequestration to a minimum extent in their objectives (policies B-F). Policy G was designed for maximizing carbon sequestration without incorporating any commercial value and therefore favored forest conservation as the most dominant management strategy. The polar opposite policy A aimed at maximum NPV, with the potential to realize this objective using a diversity of management strategies.

### Sensitivity to interest rate

**Figure 5:**
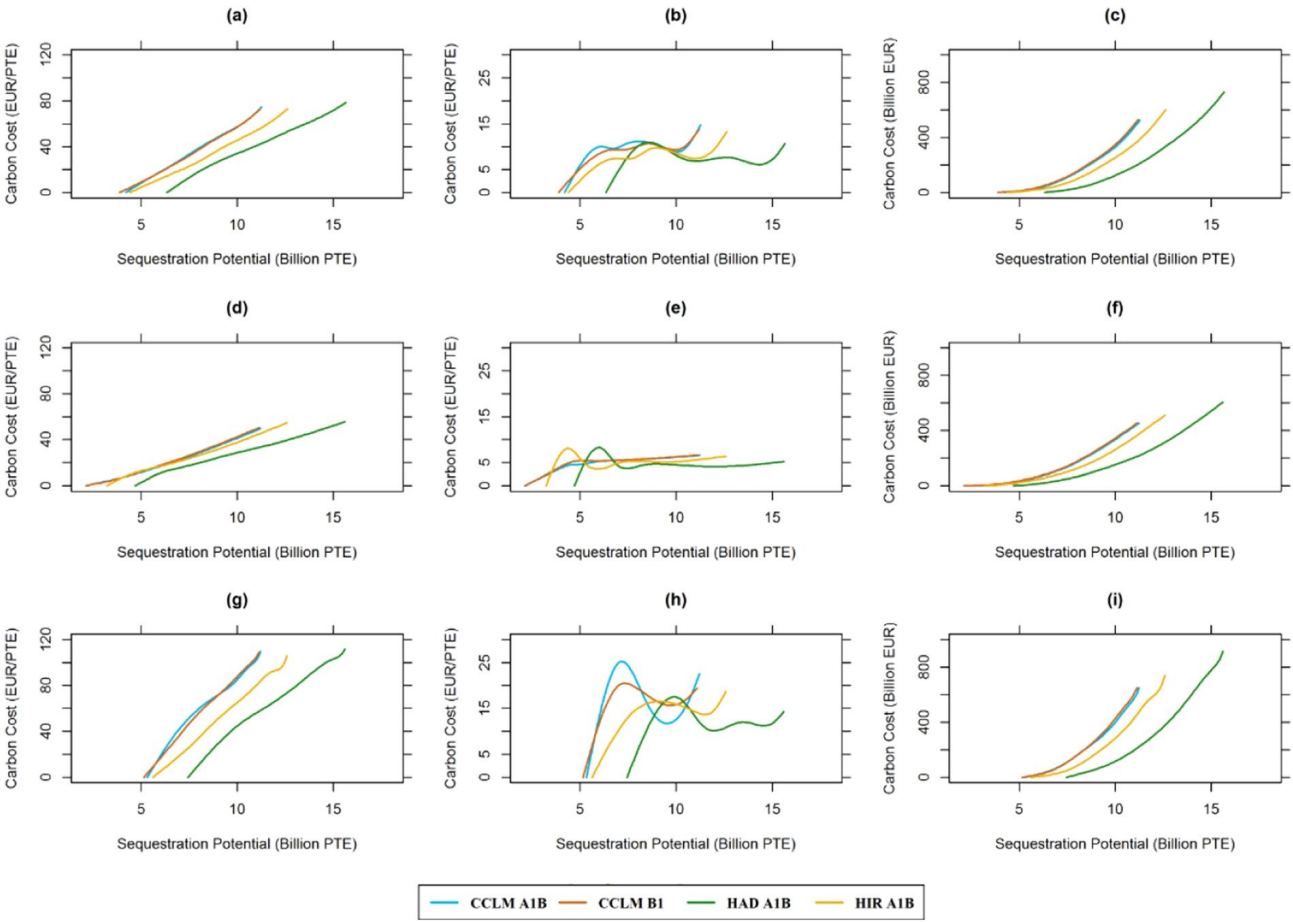
Sensitivity analysis on marginal costs of an efficient increase in carbon sequestration potentials. The figure shows the effect of interest rates on the supply, marginal and total costs for increasing the carbon sequestration potentials in Europe, with (**a**), (**b**) and (**c**) showing the marginal costs for the country-specific interest rate. Similarly, (**d**), (**e**) and (**f)** show the supply, marginal and total cost for a 2% interest rate and (**g**), (**h**) and (**i**) for a 0% interest rate.

In Fig. 5, the maximum carbon cost was reduced from 109.11 EUR/PTE for the 0% interest rate to 52.48 EUR/PTE for the 2% interest rate, whereas this was 74.59 EUR/PTE for the country-specific interest rate. The sequestration potential increased for the initial sections of the supply curve for the 0% interest rate, as the contribution of standing stock increased for the total NPV, promoting thinning regimes with lower intensity. The steepness of the supply curve increased with low interest rates and the marginal costs surpassed 20 EUR/PTE for the 0% interest rate, whereas it remained below 10 EUR/PTE for the 2% interest rate. Despite the differences on the absolute value of the marginal costs, the patterns were similar for all interest rates applied, reaching higher values at the initial sections of the supply curve. This was an indication that at low carbon price the carbon sequestration costs would increase more rapidly, proportional to the carbon sequestration. The total cost decreased with higher interest rates. The maximum total cost ranged from 647.2 to 913.1 billion EUR for the 0% interest rate and from 451.3 to 605.3 billion for the 2% interest rate. Additionally, the costs were lowest for HAD A1B climate change trajectory regardless of the interest rate applied. With increased growth rates under this trajectory, the supply curve shifted to the right, with higher carbon sequestration levels at the same carbon cost levels, compared to the other scenarios. Applying a zero discount rate for carbon (time indifferent realization of mitigation), the sequestration potential increased drastically, surpassing 20 billion PTE, however, marginal and total costs decreased (see details in Supplementary S3 3.2).

## Discussion

Besides forest growth rate as a driving ecological factor, economic factors (e.g. wood prices, harvesting costs, and discount rate) affect carbon costs^19^. We found a range of efficient solutions by balancing carbon sequestration and forest profitability, allocating the focus of CSF to countries with a high and increasing sequestration potential, e.g. Sweden^15^, and low carbon costs, e.g. Poland and Romania, and allowing for commercial wood utilization in highly valuable forests, e.g. oak species in France. For example, policy D had an estimated carbon sequestration potential of 8.6 billion PTE with a cost of 34.35 EUR/PTE, which is within the range of international recommendations^18^, especially in forestry^20,21^. Stronger climate change effects, for example according to HAD A1B and HIR A1B trajectories, would lead to an accumulation of a large amount of carbon in European forests: over 11.1 billion PTE with 41.8 EUR/PTE (Fig. 2a) because of enhanced European forest growth and productivity^15^.

Increasing frequency and intensity of disturbances under climate change was not accounted for in our modelling approach and may negatively affect the amount of sequestered carbon^22^ and counterbalance enhanced carbon sequestration figures. Seidl *et al*.^12^ have recently announced that disturbances from wind, bark beetles, and wildfires have increased in Europe’s forests throughout the twentieth century, and this can offset the effect of management strategies aiming to increase the forest carbon sink. Therefore, more detailed information about the reality of damages in European forest landscapes and management responses should be used to improve the carbon budgeting schemes in models^12^. Adopting carbon-oriented forestry might affect provisioning of other ecosystem services. For example, reducing wood harvest may increase the risk of forest disturbances and emissions in catastrophic events and thus, implementing mitigation actions should be linked to risk management programs, reducing the exposure to carbon losses. Conversely, with higher carbon density in European forests, mortality and deadwood volume would increase, leading to positive effects on biodiversity and decrease in nutrient export, favoring nutrient cycling and soil nutrient uptake^23^.

Climate Smart Forestry in Europe can only be realized by an active and multi-objective forest management strategy^14^ and may require diversification of applied management strategies^12^. To optimize CSF in our study framework, management strategies were mixed not only among species but also for a given species; because of strong dissimilarities in forest development in different countries necessitating diversified actions (details in Supplementary). Management diversity was higher for policies (B-F) weighting both NPV and PTE moderately, and extreme polices (A and G) which would shift the management towards ‘intensified’ and ‘no management’ (*in situ* carbon sequestration) strategies. These patterns were robust to time preference for NPV and PTE; however, they also affected the relative frequency of different management strategies (see Figures S5 and Fig. 5a–d in Supplementary Materials).

There are other management options in addition to increasing carbon density by controlling forest wood utilization intensity in European forests. Naudts *et al*.^7^ suggested that forest management, through wood utilization and the conversion from old growth broadleaves to conifers during the past centuries may have contributed to current global climate warming, mainly due to changes in emissivity and albedo of the forest cover. Thus, maintaining old growth broad-leaved forests might be an effective measure for enhanced climate change mitigation. However, maintaining old growth forests may lead to economic losses in Europe as these forests notably present lower growth rates and the forest industry is highly specialized in conifer timber production.

Several studies assessed leakage arising from climate change mitigation and *in situ* carbon sequestration strategies, indicating that efforts to sequester carbon in one region might result in increasing emissions elsewhere (26). However, as Gan and McCarl^24^ suggested, leakage effects might be hindered through cooperation between countries. Moreover, analyzing the life cycle of wood products to quantify their substitution effects for energy-intensive materials and fossil fuels may highlight the further positive effects of forest sector for carbon mitigation^20^. Ideally, this point should be considered in the studies of forest adaptation and mitigation under climate change, providing a holistic analysis of mitigation options. Additionally, further research may include the ecological response of forest ecosystems to changes in environmental conditions^22^ and the short and long term adaptive measures necessary to establish resilient ecosystems in the portfolio of CSF management options^12^. Integration of other non-commercial European tree species, especially from Mediterranean area, would improve forest carbon cycle analysis and homogenous representativeness of the results for the entirety of Europe.

We obtained maximum carbon costs ranging from 50.77 to 143.49 EUR/PTE, compatible with the values found in international studies^20,25^. The shape of the response curve of carbon cost in European forestry was similar to Lubowski *et al*.^21^. The authors estimate an uptake of approximately 225 million tons/year carbon for a higher carbon cost of US$60.00 /ton. Our study suggests higher costs in Europe, with sequestration levels from 120 to 159 million PTE/year at a $60.00 (1 euro = 1.1 dollars) cost level, depending on the climate change scenarios. Numerous studies evaluating social carbon costs indicate similar patterns, with reported social costs higher than current carbon costs (e.g. ranging from $25 to $296/tC on average)^26^. The maximum carbon sequestration scenarios for our study would be able to store 11% and 15% (12.4% on average) of current annual GHG emissions in the European Union (4678.8 million tons of CO_2_ equivalent^27^) under the lower bound emission scenario (CCLM B1), and under the up/bound sequestration scenario (HAD A1B), respectively. Nevertheless, findings of this type are sensitive to economic conjuncture, e.g. discount rate, and future climatic conditions- both affecting the magnitude of carbon sequestration and the costs of different climate policies. In this study, prices, and costs were assumed to be exogenous to the forestry system. To overcome this issue, forest and wood sector models should be linked to the forest production system (supply side) to internally define the changes in price and costs endogenously.

CSF as applied in this study may achieve, for example, 48% of the total sequestration potential with 78% lower total costs compared to the maximum carbon sequestration. However, the carbon cost in Europe might impose a great obstacle to significantly increase the sequestration *in situ*, as the prices of the Emissions Trading System (EU ETS) decreased from 30.00 EUR/ton in 2008 to 7.70 EUR/ton of CO_2_ in 2015 (110 and 28.23 EUR/ton of carbon respectively). At current price levels, the additional carbon sequestration considering all countries and species included in our study would increase on average from 4.7 Billion PTE (58.8 Million PTE/year) to 7.9 Billion PTE (98.4 Million PTE/year), corresponding to 7.7% of European GHG emissions (Fig. 2).

## Methods

### Data and Simulation

We examined outputs from model simulations for a network of 132 intensively monitored forest plots, distributed over 18 European countries, covering ten environmental zones (see Reyer *et al*.^28^ for more details on the plot selection and environmental zones). The main central European tree species included in our study were *Picea abies, Pinus sylvestris, Fagus sylvatica, Quercus petraea* and *Quercus robur*. These species make up the backbone of commercial forestry in Europe^13^; however, they also sequester a substantial amount of carbon due to their fast growth and the extent of their habitat in Europe^28^. We calculated the economic outcomes of four forest management regimes, including forest conservation (no management activities where stands develop naturally), BAU, intensified and decreased forest wood harvesting rate, for different species and European countries. Management regimes, meaning harvesting trees from upper, middle, and lower canopy layers (from above, middle, and below thinning regimes, respectively) were assigned according to the tree species (e.g. from above for *Quercus spp*. and from below for *Piceas abies*). For economic performance evaluation purposes, we derived the harvesting revenues and costs for each species in each country from the EFISCEN model database^29^ and the long-term interest rates reported by the European Central Bank.

We simulated the responses of forest growth and carbon budget to climate for a duration of 80 years, from 2011 to 2090. The simulations included permutations of the four management interventions in three decision points (2020, 2030, and 2040), thus yielding 64 management regimes. The simulations were subject to four climate change trajectories based on three regional climate model- general circulation model combinations for the A1B scenario, and a model for the B1 scenario. All trajectories resemble the current representative carbon concentrations of 4.5 and 6.0^30^ to model climate development pathways in the 21st century, representing roughly the medium range of global warming (2.6–3.8 °C in 2080–2099 compared to 1900–1919) expected under current mitigation policy pledges^16,31^.

### Process-based forest model 4C

We applied the model 4 C for analyzing forest responses in terms of wood production and carbon sequestration for different species in Europe and different climate change conditions. 4 C is a process-based forest model that simulates forest responses depending on climatic conditions. The model is capable of simulating forest structure, LAI, carbon and water balance, as well as various management interventions including harvesting and thinning^32^ in pure and mixed stands. The essential elements in the soil carbon and nitrogen cycle are the litter and dead fine roots, which supply the soil with organic matter, litter and root turnover and the nitrogen uptake by plants. The 4 C model describes processes at tree- and stand-level based on eco-physiological experiments, long term observations and physiological modeling (see S1-Fig. 4). Trees of similar dimensions are aggregated into cohorts and their growth and mortality are explicitly modeled for each cohort at the stand level assuming horizontal homogeneity within cohorts. The model (4 C) has been evaluated across Europe using long-term forest growth data as well as eddy-covariance flux measurements^28^. We applied 4 C to examine the response of forest ecosystems to climate change in terms of the total carbon budget in the ecosystem, wood productivity, and harvesting volume. While 4 C includes drought stress^33^, it does not include the effects of disturbances such as wind, fire, and insects. The total carbon in the ecosystem represents a sum of the total carbon in biomass (above- and below-ground) and the total carbon in soil. We scaled the plot-level simulations of 4 C to the country level by using species specific coverage area and assuming representativeness of the simulated plots for the entire coverage area.

### Economic evaluation

We evaluated changes in forest management for climate change mitigation during the 21^st^ century. Economic impacts from adoption of a mitigation management strategy were evaluated in terms of NPV, applying a similar approach as in Hanewinkel *et al*.^13^. The NPV equation (1) was calculated using the EFISCEN model price data for each country and species in the data set (see details in Supplementary 1) and the long-term interest rate defined by the European Central Bank for each country to account for regional differences in economy.

1 $$NPV=\frac{Fs}{{(1+i)}^{T-1}}+\sum _{t=1}^{T}\frac{T{r}_{t}}{{(1+i)}^{t-1}}-Is$$

where:

*NPV*: Net Present Value

*Fs*: net stumpage value of stock at the end of the simulation period

*T*: simulation period

*i*: interest rate

*Tr*_*t*_: discounted net revenue generated by thinning in year *t*

*Is*: net stumpage value of stock at the beginning of the simulation period.

The NPV (equation (1)) was defined based on the difference between the discounted standing stock values at the beginning (*Is*) and at the end of simulation period (*Fs*) and the net harvesting revenues (*Tr*).

We calculated the costs per ton of carbon for each species and country included in the study (see Fig. 1) using the discounted carbon method. The discounted amount of carbon is usually referred to as present tons equivalent (PTE). According to Boyland^17^, discounting the carbon has two main functions: 1- to account for carbon sequestration fluctuations that may occur during the planning horizon and 2- to account for the urgency carbon sequestration in early periods rather than at the end of the planning horizon. The carbon sequestered for each management scenario was calculated applying equation (2) by summing the discounted yearly carbon sequestered during the simulation period, with a 2% discount rate.

2 $${C}_{i}=\sum _{t=1}^{T}\frac{(Cto{t}_{t}-Cto{t}_{t-1})}{{(1+i)}^{t-1}}$$

where:

*C*_*i*_: discounted sequestered carbon for management *i* (PTE/ha)

*Ctot*_t_: total carbon in the ecosystem at year *t*

*i*: discount rate.

The carbon sequestration costs per PTE were calculated according to equation (3). Initially, we defined the management regimes yielding maximum NPV and the respective carbon sequestration level for this management alternative. The carbon sequestration cost was computed based on the ratio of the NPV reduction and the increase in carbon sequestration yielded by a given management alternative for each climate change trajectory, species and country. The NPV reduction was defined as the difference in the NPV achieved by management for maximum NPV, and the NPV yielded by the considered management alternative (numerator of equation (3)). The increase in carbon sequestration was computed as the difference in carbon sequestration between the considered management, and carbon sequestration obtained through the maximum NPV management (denominator of equation (3)).

3 $$C{C}_{m}=\frac{(MaxNP{V}_{ijk}-NP{V}_{ijkm})}{({C}_{ijkm}-CMaxNP{V}_{ijk})}$$

where:

*CC*_*m*_: carbon cost of mitigation strategy *m* (EUR/PTE)

*MaxNPV*_*ijk*_: maximum NPV under climate change *i* for species *j* and country *k* (EUR/ha)

*C*_*maxNPV*_: discounted carbon sequestered by the management generating the maximum NPV (PTE/ha)

*NPV*_*ijkm*_: NPV generated under climate change *i* for species *j* and country *k* through mitigation strategy *m* (EUR/ha)

*C*_*ijkm*_: discounted carbon under climate change *i* for species *j* and country *k* sequestered through mitigation strategy *i* (PTE/ha).

### Balancing carbon mitigation and forest profitability for Climate Smart Forestry

Forest management usually involves the coordination of a series of activities and processes competing for the same resources. For these types of complex problems with competing forest management objectives, goal programming (GP) is especially useful^34^. One of the most common goal programming approaches is the WGP (Weighted Goal Programming). In WPG, undesirable deviations from the goals are weighted and included in the objective function to be minimized^35^. According to Buongiorno & Gilles^36^, the weighting procedure has two purposes in GP models: to express all objectives in a comparable scale and to express the relative importance of each objective.

In our study, we assumed that carbon sequestration and economic profitability are competing goals for forest managers as in van Kooten *et al*.^37^; thus, WGP is a suitable tool to achieve a balanced solution, allowing selection of management regimes while simultaneously considering NPV maximization and carbon sequestration. We established a trade-off curve of NPV and carbon sequestration, i.e. the Pareto frontier of NPV and carbon sequestration. The weights of both objectives were varying for each species, country, and climate change trajectory through the global optimum solutions of the WGP model. The deviations from maximum NPV and maximum carbon sequestration for Europe were the basis for WGP, i.e. to minimize deviations. Therefore, our approach is equivalent to a compromise programming model on the *L*_1_-norm. The WGP model is given by:

4 $$MinZ={w}_{1}\,.\sum _{i=1}^{C}\frac{NP{{V}_{i}}^{-}}{Norm{N}_{i}}+\,{w}_{2}\,.\sum _{i=1}^{C}\frac{CA{{R}_{i}}^{-}}{Norm{C}_{i}}$$

s. t.

5 $$\sum _{j=1}^{S}\sum _{k=1}^{P}\sum _{l=1}^{N}{n}_{ijkl}{x}_{ijkl}+NP{{V}_{i}}^{-}=MaxNP{V}_{i}\quad \quad \forall i$$

6 $$\sum _{j=1}^{S}\sum _{k=1}^{P}\sum _{l=1}^{N}{c}_{ijkl}{x}_{ijkl}+CA{{R}_{i}}^{-}=MaxCA{R}_{i}\quad \quad \forall i$$

7 $$\sum _{l=1}^{N}{x}_{ijkl}=1\quad \quad \forall i,\forall j,\forall k$$

8 $${x}_{ijkl}\in \{0,1\}\quad \quad \forall i,\forall j,\forall k,\forall l$$

where:

*C*: set of climate change scenarios

*S*: set of species

*P*: set of countries

N: set of management regimes

*w*_1_: weight for the NPV

*w*_2_: weight for the carbon sequestration

*x*_*ijkl*_: a binary variable that takes value 1 case management *l* is selected under climate change *i* for species *j* and country *k* or value 0 otherwise

*n*_*ijkl*_: net present value generated by management *l* under climate change *i* for species *j* and country *k* (EUR/ha)

*c*_*ijkl*_: carbon sequestered by management *l* under climate change *i* for species *j* and country *k* (t of C/ha)

*MaxNPV*: maximum NPV under climate change *i* (EUR/ha)

*MaxCAR*: maximum carbon sequestered under climate change *i* (t of C/ha)

$NP{V}_{i}^{-}$: deviation in NPV for compared to the maximum NPV management under climate change *i* (EUR/ha)

$CA{R}_{i}^{-}$: deviation in carbon sequestration compared to the maximum carbon sequestration management under climate change *i* (t of C/ha)

*NormN*_*i*_: NPV normalization constant for under climate change *i*

*NamC*_*i*_: carbon sequestration normalization constant for under climate change *i*.

The objective function (equation (4)) aimed to minimize the sum of negative deviations, i.e. to decrease on both the NPV and carbon sequestration, for all countries and species in the remaining time of 21^st^ century. The weights *w*_1_ and *w*_2_ were defined as the relative importance of NPV and carbon sequestration defining different forest policy schemes to implement CSF by favoring NPV over PTE or vice versa. The deviations were expressed in relative terms for each climate change scenario, applying a normalizing constant representing the objective range. Constraint equation (5) related the NPV deviations for all countries and species with the respective maximum value, established through the management regimes yielding the highest NPV. Constraint equation (6), similarly to constraint equation (5), related the carbon sequestration deviations of all species and countries to its maximum value. Constraint equation (7) assured that only one management regime is selected for each species and country. Constraint equation (8) guarantees that the variables *x*_*ijkl*_ assume only binary values.

The optimization model was solved multiple times, varying the weights for carbon sequestration and NPV from 0 to 1 in a 0.1 interval by using the software LINGO 13.0 on a PC with Intel® Core™ i5-3337U CPU, with 1.80 GHz and 4GB of memory.

Finally, the WGP optimization procedure was repeated to analyze the sensitivity of obtained optimization results to policy schemes for mitigation, maximum carbon sequestration potential, and economy-based efficient carbon sequestration, and to varying interest rates (0%, the long-term interest rates reported by the European Central Bank, and 2%). A detailed explanation of the sensitivity analysis and the outcomes can be found in the Supplementary Information.

### Availability of materials and data

The datasets generated during and/or analyzed during the current study are available from the corresponding author on reasonable request.

## Electronic supplementary material

Supplementary Info 1

Supplementary Info 2

Supplementary Info 3
