# Supplementary material for: Realizing Mitigation Efficiency of European Commercial Forests by Climate Smart Forestry
Source: Sci Rep. 2018 Jan 10;8:345. doi: 10.1038/s41598-017-18778-w (PMC5762874; doi:10.1038/s41598-017-18778-w)
Supplement: Supplementary file 1 — Supplementary Info 1 [file 41598_2017_18778_MOESM1_ESM.pdf]

Title page

Supplementary 1: Data

Title: *Realizing Mitigation Efficiency of European Commercial Forests by Climate Smart Forestry*

Authors: Rasoul Yousefpour[1]*, Andrey Lessa Derci Augustynczik[1], Christopher P.O. Reyer[2], Petra Lasch-Born[2], Felicitas Suckow[2], and Marc Hanewinkel[1]

[1] Chair of Forestry Economics and Forest Planning, Faculty of Environment and Natural Resources, University of Freiburg, Tennenbacherstr. 4, D-79106 Freiburg

[2] Potsdam Institute for Climate Impact Research (PIK), Telegraphenberg A62/1.05, D-14412 Potsdam

*Corresponding author (E-mail: rasoul.yousefpour@ife.uni-freiburg.de, Tel: +49-761-2033688, Fax: +49-761-2033690)

Data

To analyze forest growth across Europe and management outcomes, we applied the stand-scale process-based forest model 4C (http://www.pik-potsdam.de/4c/). We examined outputs from 4C model simulations covering a network of 132 intensively monitored forest plots, distributed over 18 European countries and 10 environmental zones (S1-Figure 1, see Reyer et al.[1] for more details on the plot selection and environmental zones. The main central European tree species included in our study were: *Fagus sylvatica, Picea abies, Pinus sylvestris, Quercus petraea* and *Quercus robur*. The forest data for each plot (e.g. tree species, age, stem number, diameter at breast height and height) were used to initialize the 4C model, which was then modified by the climate scenarios. Soils were initialized from a horizontal description of soil physics and chemistry as described in Reyer et al.[1].

The plot distribution applied in our study was not homogeneous, with lower sampling density in Mediterranean areas and South-Eastern Europe, representing majorly forests in higher elevations. Nevertheless, as we applied a country and species-specific approach, our analysis was performed exclusively for sampled species. Moreover, the simulated forest development of the plots in Mediterranean areas obtained through 4C is compatible with values reported in the literature [2,3,4].

In order to calculate the economic outcomes of forest management for the various species and countries we derived the harvesting revenues and costs for each species in each country from the EFISCEN model database. We considered static wood prices during the simulation period, given the stability of real sawn wood prices in Europe during the past 30 years[5]. Forest areas were derived from the evaluation performed by Brus et al.[6], the EFISCEN inventory database and national forest inventories of each country. Tables 1, 2, and 3 illustrate the allocation of plots, coverage area, and prices to different European countries and tree species, respectively.

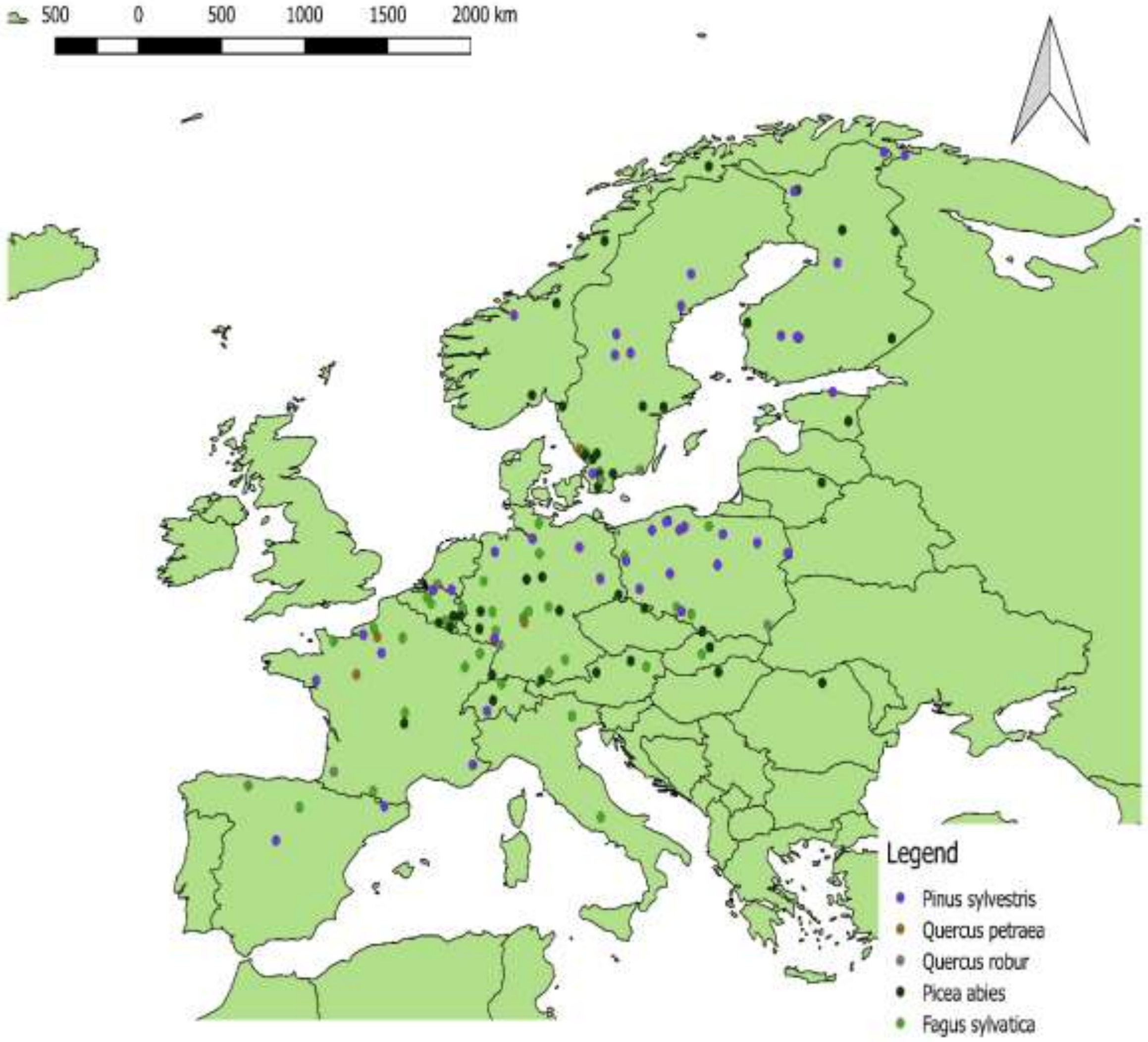

S1-Figure 1. Map of forest plots used in this study. The model 4C was initialized for each plot. *Figure 1 was created with QGIS Version 2.18.13 (http://www.qgis.org/en/site/). The base map was made with Natural Earth. Free vector and raster map data @ naturalearthdata.com.*

S1-Table 1. Number of plots for each species and country.

Country	*Acronym*	*Fagus sylvatica*	*Picea abies*	*Pinus sylvestris*	*Quercus petraea*	*Quercus robur*
Austria	AU	1	2	-	-	-
Belgium	BL	4	4	1	-	-
Czech Republic	CZ	-	1	-	-	-
Estonia	EE	-	1	1	-	-
Finland	SF	-	6	6	-	-
France	FR	7	1	4	2	1
Germany	DL	11	8	5	3	1
Hungary	HU	-	1	-	-	-
Italy	IT	2	-	-	-	-
Lithuania	LT	-	1	-	-	-
The Netherlands	NL	-	-	1	-	1
Norway	NO	-	5	2	-	-
Poland	PL	4	1	13	-	1
Romania	RO	-	1	-	-	-
Slovak Republic	SR	1	1	-	-	-
Spain	ES	1	-	2	-	1
Sweden	SW	3	8	6	1	2
Switzerland	SZ	1	1	1	-	-
Total		35	42	42	6	7

S1-Table 2. Forest areas by species in ha and its respective percentage related to the total forest area in the country.

Country	*Fagus sylvatica*	*Picea abies*	*Pinus sylvestris*	*Quercus petraea*	*Quercus robur*
Austria	368,410 (9.5%)	2,319,044 (59.9%)	-	-	-
Belgium	7,491 (1.1%)	184,705 (27%)	43,095 (6.3%)	-	-
Czech Republic	-	1,365,275 (51.2%)	-	-	-
Estonia	-	398,588 (17.9%)	626,800 (28.1%)	-	-
Finland	-	5,140,677 (23.1%)	12,969,772 (58.4%)	-	-
France	182,638 (1.1%)	57,560 (0.3%)	167,728 (1%)	1,631,000 (9.6%)	2,107,000 (12.4%)
Germany	1,399,659 (12.3%)	3,232,636 (28.3%)	2,791,347 (24.4%)	383,635 (3.4%)	488,263 (4.3%)
Hungary	-	27,166 (1.3%)	-	-	-
Italy	515,597 (5.5%)	-	-	-	-
Lithuania	-	407,110 (18.7%)	-	-	-
The Netherlands	-	-	100,380 (26.7%)	-	28,029 (7.5%)
Norway	-	1,275,592 (10.5%)	861,322 (7.1%)	-	-
Poland	388,913 (4.1%)	487,282 (5.2%)	6,083,981 (64.5%)	-	313,845 (3.3%)
Romania	-	1,494,100 (21.8%)	-	-	-
Slovakia	577,997 (29.8%)	511,340 (26.4%)	-	-	-
Spain	486,000 (2.6%)	-	1,184,000 (6.4%)	-	257,040 (1.4%)
Sweden	94,231 (0.3%)	7,616,000 (27.1%)	10,976,000 (39.1%)	75,307 (0.3%)	95,846 (0.3%)
Switzerland	221,494 (17.7%)	475,847 (37.9%)	35,293 (2.8%)	-	-

S1-Table 3. Wood prices (Net wood prices in EUR/m³)

Country	*Fagus sylvatica*	*Picea abies*	*Pinus sylvestris*	*Quercus petraea*	*Quercus robur*
Austria	30.39	30.57	-	-	-
Belgium	17.34	11.24	8.07	-	-
Czech Republic	-	24.89	-	-	-
Estonia	-	18.33	15.47	-	-
Finland	-	25.23	21.04	-	-
France	34.61	23.25	22.68	42.15	42.15
Germany	26.92	21.15	14.56	42.15	42.15
Hungary	-	15.78	-	-	-
Italy	20.67	-	-	-	-
Lithuania	-	13.88	-	-	-
The Netherlands	-	-	12.84	-	35.47
Norway	-	27.47	21.25	-	-
Poland	13.77	18.85	19.40	-	16.27
Romania	-	9.48	-	-	-
Slovak Republic	12.49	16.61	-	-	-
Spain	18.23	-	19.09	-	18.225
Sweden	16.22	21.64	20.54	16.22	16.22
Switzerland	43.38	28.36	28.36	-	-

Long term interest rates

S1-Table 4 shows the long-term interest rates reported by the European Central Bank as an average of the long-term interest rates from November/2014 to November/2015. The data for Norway and Switzerland was retrieved from the OECD database.

S1-Table 4. ECB long term interest rates for European countries[7,8]

Long-term interest rates (%)					
Country	Interest rate	Country	Interest rate	Country	Interest rate
Austria	0.78	Germany	0.54	Poland	2.66
Belgium	0.89	Hungary	3.53	Romania	3.52
Czech Rep.	0.66	Italy	1.86	Slovakia	1.01
Estonia*	1.14	Lithuania	1.53	Spain	1.80
Finland	0.75	Netherlands	0.74	Sweden	0.80
France	0.89	Norway	1.58	Switzerland**	0.17

*No equivalent of long-term interest rate available. The interest applied corresponds to the long-term interest rate in Latvia.

** The interest rate represents the average of the period May/2014 to November/2015

Climate

The climate change scenarios were taken from the following combinations of regional climate models and general circulation models: CCLM/ECHAM5 (CCLM_A1B), HadRM3/HadCM3 (HAD_A1B) and HIRHAM3/Arpège (HIR_A1B) driven by the A1B emission scenario. This emission scenario assumes a medium-high emission level, with economic growth, rapid technology development, and a material-intensive lifestyle[9] so that a climate target of maximum 2°C global surface temperature warming at the end of century is exceeded. In addition, we used the CCLM/ECHAM5 model driven by the B1 emission scenario (CCLM_B1) whose GCM forcing corresponds nearly to the 2°C target. The B1 emission scenario considers changes in economic structures, with reduction in material intensity and introduction of resource-efficient technologies[9]. S1-Figure 2 shows the timeline of the average temperature anomalies of the four climate scenarios for the plots in each country and S1-Figure 3 illustrates the pathway of CO_2 concentration increase for both A1B and B1 scenarios over time (2010-2090).

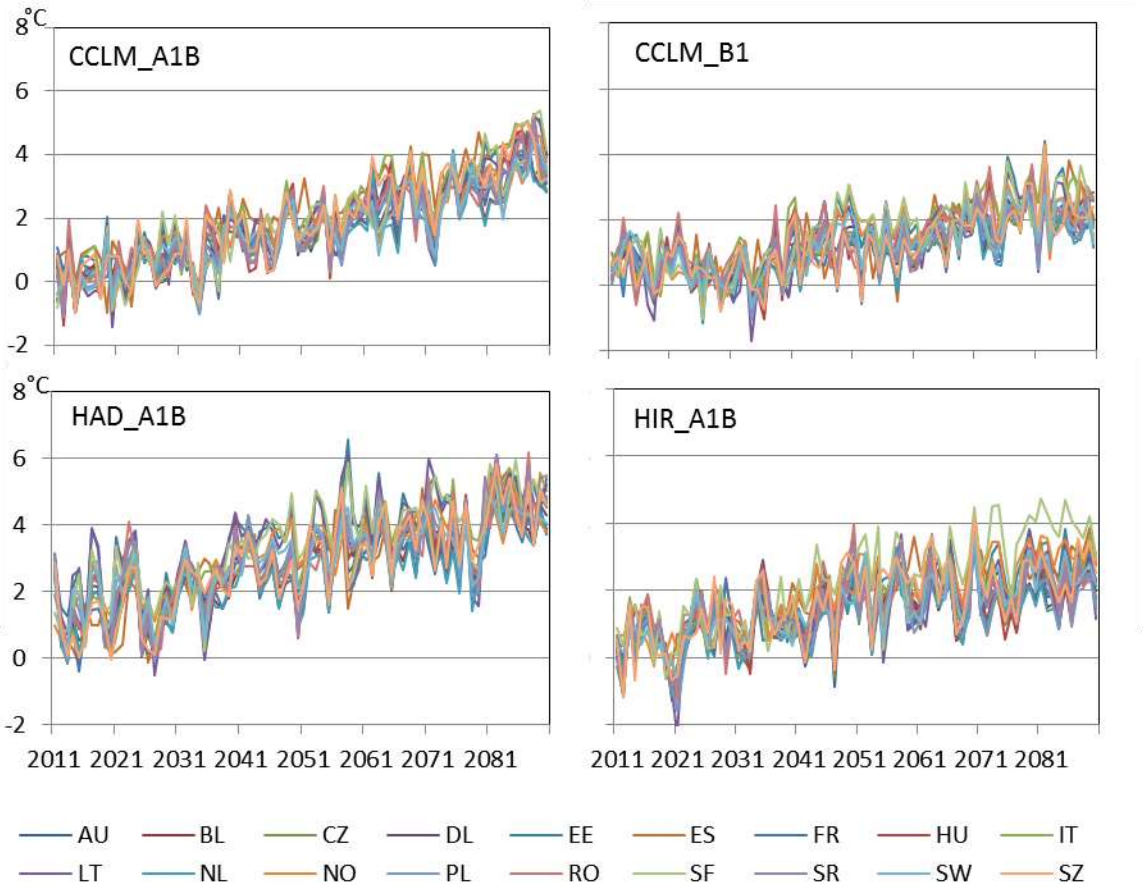

S1-Figure 2. Anomalies of temperature, relative to 1971-2000, for all plots in each country and the four climate scenarios. The plots of each country are averaged /year. The acronyms of the countries are listed in S1-Table 1

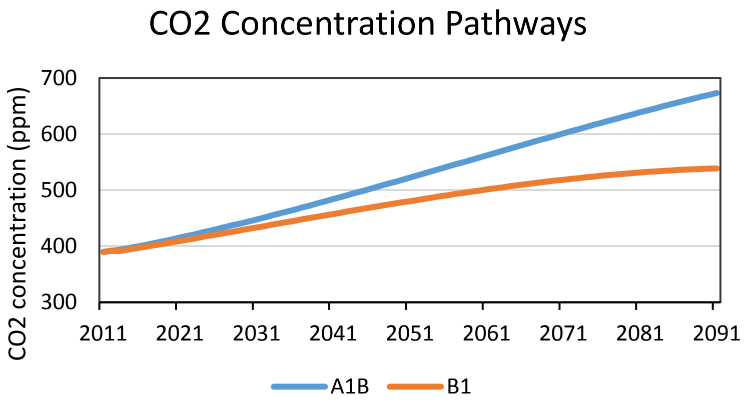

S1-Figure 3. CO2 concentrations of A1B and B1 scenarios in the atmosphere over simulation period 2010-2090.

The 4C model

Process-based forest models describe forest dynamics in detail, based on controlling processes at tree, soil and atmosphere level[10,11]. Given the capacity of these models to capture carbon, nitrogen and water cycles, they are most suitable to assess the forest ecosystems responses to new climatic conditions[12]. Here, we applied the process-based model 4C (http://www.pik-potsdam.de/4c/), for analyzing forest responses in terms of wood production and carbon sequestration for different species in Europe and under different climate change scenarios.

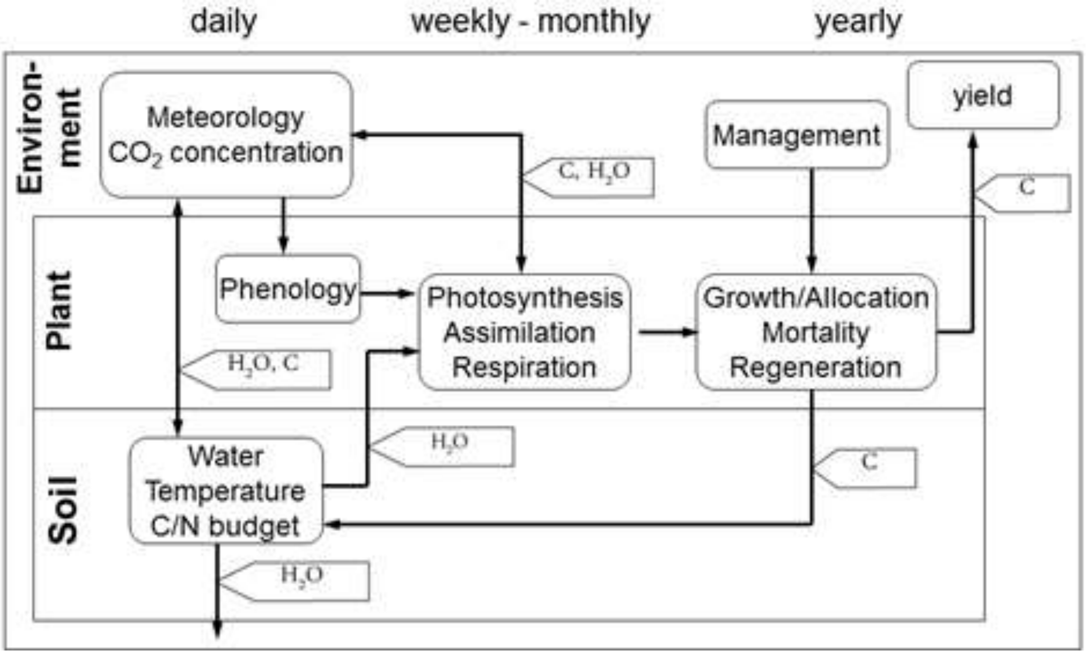

S1-Figure 4. Scheme of the model 4C with the main processes, fluxes and pools considered in this study.

4C is a process-based forest model that simulates forest responses to changing climatic conditions. The model is capable of simulating forest structure, LAI, carbon and water balance and various management interventions, including harvesting, thinning and regeneration[13]. It describes processes on tree- and stand-level based on eco-physiological experiments, long term observations and physiological modeling (see S1-Fig. 4). The trees of the forest stand are aggregated into cohorts and their establishment, growth and mortality are explicitly modeled for each cohort on the stand level assuming horizontal homogeneity whithin each cohort.

The annual course of net photosynthesis was simulated with a mechanistic formulation of net photosynthesis as a function of environmental influences (temperature, water and nitrogen availability, radiation and CO2). The physiological capacity (maximal carboxylation rate) was calculated on the basis of optimization theory (modified after Haxeltine and Prentice[14]), plus calculation of total tree respiration following the concept of constant annual respiration fraction as proposed by Landsberg and Waring[11]. The allocation pattern of annual net primary production (NPP) to the tree organs and tree growth were modeled with a combination of pipe model theory[15], the functional balance hypothesis[16] and several allometric relationships extended to respond dynamically to water and nutrient limitations. The start and end of the vegetation period were estimated as functions of air temperature and length of the day[17]. Water and nitrogen availability, which affect growth and mortality of trees, depend on the soil parameters, the climatic conditions, and the stand development. Mortality depends explicitly on the carbon balance of the tree cohorts and failure to reproduce foliage over several years increases mortality probability. Mortality due to disturbances has not yet been modeled. The water balance was calculated from potential evapotranspiration according to Turc/Ivanov[18], interception, and percolation Transport of water in the multi-layered soil was calculated with a daily time step[19] by a simple percolation model and controlled by a model-specific water conductivity parameter[20, 21] depending on the soil texture. Root uptake is limited by the transpiration demand of all trees and the plant available water. The tree cohorts compete for water and nitrogen and satisfy their demand layer by layer through their fine roots in proportion to the fine root mass of all cohorts in the respective layer.

Currently, the model is parameterized for the five most abundant tree species of Central Europe (beech (*Fagus sylvatica* L.), Norway spruce (*Picea abies* L. Karst.), Scots pine (*Pinus sylvestris* L.), oaks (*Quercus robur* L., and *Quercus petraea* Liebl.), and birch (*Betula pendula* Roth)) as well as for other tree species. We applied 4C to examine the response of forest ecosystems to climate change, in terms of the total carbon budget in the ecosystem, wood productivity, and harvesting volume. The total carbon in the ecosystem represents a sum of the total carbon in biomass (above and belowground) and the total carbon in soil. Although the development of *Quercus robur* and *Quercus petraea* were modelled identically in 4C, the economic analysis for these species was performed separately due to the distinct wood prices and forest cover in each country. Thus, country-specific conditions for the implementation of mitigation strategies by sequestering carbon in forest biomass were defined.

4C has been evaluated across Europe using long-term forest growth data eddy-covariance flux measurements, daily transpiration and soil water content as well as a long-term dataset on annual tree ring increments at different temporal scales[1, 22, 23, 24, 25].

Response to climate change

The impacts of climate change on forest productivity forecasted by 4C are extremely positive in Europe (Reyer et al.[1]). Changes in NPP forecasted by 4C, according to Reyer et al.[1] show that under increasing CO_2 concentration (Persistent CO_2 effects), forest productivity

increases across Europe, especially for Boreal regions. At the species level, the model indicates a NPP change of 0 to 0.4 Mg of C/year for *Picea abies*, -0.2 to 3.9 Mg of C/year for *Pinus sylvestris*, -0.5 to 3.1 Mg of C/year for *Fagus sylvatica* and -0.9 to 3.7 Mg of C/year for *Q. petraea* and *Q. robur*[1]. With constant CO_2 concentration (Acclimation CO_2 effects) the climate change impacts are predominantly positive in Scandinavia, with mixed effects in central Europe, whereas in Mediterranean areas negative impacts were observed.

References

1. Reyer, C. et al. Projections of regional changes in forest net primary productivity for different tree species in Europe driven by climate change and carbon dioxide. *Annals of Forest Science* **71,** 211-225 (2014)
2. Condés, S. et al. Mixing effect on volume growth of Fagus sylvatica and Pinus sylvestris is modulated by stand density. *Forest Ecology and Management* **292**, 86-95 (2013).
3. Sabaté, S. et al. Likely effects of climate change on growth of *Quercus ilex, Pinus halepensis, Pinus pinaster, Pinus sylvestris and Fagus sylvatica* forests in the Mediterranean region. *Forest Eecology and Management* **162,** 23-37 (2002).
4. Tegel W, et al. A recent growth increase of European beech (*Fagus sylvatica* L.) at its Mediterranean distribution limit contradicts drought stress. *European Journal of Forest Research* 133:61-71 (2014).
5. De Groot, H. L. F. et al. Mapping Resource Prices: The Past and the Future (European Commission - DG Environment (ENV.G.1/FRA/20410/0044), Rotterdam, 2012)
6. Brus DJ, et al. Statistical mapping of tree species over Europe. *European Journal of Forest Research* **131,** 145-157 (2013).
7. ECB (European Central Bank). Statistics - Long-term interest rate statistics for EU Member States. https://www.ecb.europa.eu/stats/money/long/html/index.en.html. Access: 08/12/2015 (2015).
8. OECD (Organisation for Economic Co-operation and Development). Monthly Monetary Financial Statistics. Available: https://stats.oecd.org/index.aspx?queryid=6779. Access: 08/12/2015 (2015).
9. Nakicenovic, N. Greenhouse gas emissions scenarios. *Technological Forecasting and Social Change* **65,** 149-166 (2000).
10. van Oijen, M. et al. Process-based modeling of timothy regrowth. *Agronomy Journal* **97,** 1295-1303 (2005).
11. Landsberg, J. J. & Waring, R. H. A generalized model of forest productivity using simplified concepts of radiation-use efficiency, carbon balance and partitioning. *Forest Ecology and Management* **95,** 209-228 (1997).
12. Morales, P. et al. Comparing and evaluating process-based ecosystem model predictions of carbon and water fluxes in major European forest biomes. *Global Change Biology* **11,** 2211-2233 (2005).
13. Lasch-Bonn, P. et al. Model-based analysis of management alternatives at stand and regional level in Brandenburg (Germany). *Forest Ecology and Management* **207,** 59-74 (2005).
14. Haxeltine, A. & Prentice, I. C. BIOME3: An equilibrium terrestrial biosphere model based on ecophysiological constraints, resource availability, and competition among plant functional types. *Global Biogeochemical Cycles* **10,** 693-709 (1996).
15. Shinozaki, K. et al. A quantitative analysis of plant form - the pipe model theory. I. Basic analysis. *Japanese Journal of Ecology* **14,** 97-105 (1964).
16. Davidson, R. L. Effect of root/leaf temperature differentials on root/shoot ratios in some pasture grasses and clover. *Ann. Bot.* **33,** 561-569 (1969).
17. Schaber, J. & Badeck, F. W. Physiology-based phenology models for forest tree species in Germany. *International Journal of Biometeorology* **47,** 193-201 (2003).
18. Dyck, S. & Peschke, G. Grundlagen der Hydrologie. (Verlag für Bauwesen GmbH, Berlin, 2005).

19. Grote, R. & Suckow, F. Integrating dynamic morphological properties into forest growth modelling. I. Effects on water balance and gas exchange. *Forest Ecology and Management* **112,** 101-119 (1998).
20. Glugla, G, Berechnungsverfahren zur Ermittlung des aktuellen Wassergehaltes und Gravitationswasserabflusses im Boden. *Albrecht-Thaer-Archiv* **13,** 371-376 (1969).
21. Koitzsch, R. Schätzung der Bodenfeuchte aus meteorologischen Daten, Boden- und Pflanzenparametern mit einem Mehrschichtmodell. *Z. f. Meteor.* **27,** 302-306 (1977).
22. Gutsch, M. et al. Modeling of two different water uptake approaches for mono- and mixed-species forest stands with 4C – Model validation across scales. *Forests* 6, 2125-2147 (2015).
23. Borys, A. et al. The impact of climate change under different thinning regimes on carbon sequestration in a German forest district. *Mitigation and Adaptation Strategies for Global Change* **21,** 861-881 (2014).
24. van Oijen, et al. Bayesian calibration, comparison and averaging of six forest models, using data from Scots pine stands across Europe. *Forest Ecology and Management* **289,** 255-268 (2013).
25. Reyer, C. et al. Integrating parameter uncertainty of a process-based model in assessments of climate change effects on forest productivity. *Climatic Change* 137, 395–409 (2016).
