# Supplementary material for: Realizing Mitigation Efficiency of European Commercial Forests by Climate Smart Forestry
Source: Sci Rep. 2018 Jan 10;8:345. doi: 10.1038/s41598-017-18778-w (PMC5762874; doi:10.1038/s41598-017-18778-w)
Supplement: Supplementary file 2 — Supplementary Info 2 [file 41598_2017_18778_MOESM2_ESM.pdf]

Title Page

Supplementaries 2: Management strategy

Title: Realizing Mitigation Efficiency of European Commercial Forests by Climate Smart Forestry

Authors: Rasoul Yousefpour[1]*, Andrey Lessa Derci Augustynczik[1], Christopher P.O. Reyer[2], Petra Lasch-Born[2], Felicitas Suckow[2], and Marc Hanewinkel[1]

[1] Chair of Forestry Economics and Forest Planning, Faculty of Environment and Natural Resources, University of Freiburg, Tennenbacherstr. 4, D-79106 Freiburg

[2] Potsdam Institute for Climate Impact Research (PIK), Telegraphenberg A62/1.05, D-14412 Potsdam

*Corresponding author (E-mail: rasoul.yousefpour@ife.uni-freiburg.de, Tel: +49-761-2033688, Fax: +49-761-2033690)

Management strategy

To analyze the effects of forest utilization intensity on the carbon density, four options were included as forest management strategies as defined below:

1- Forest conservation (no thinning interventions are performed and stands develop unmanaged);
2- Business as usual (BAU) management (thinning regimes are implemented as the current forest management guidelines in Europe recommend and differentiated among species according to S2-Table 1);
3- Adaptive management with intensified forest wood harvesting;
4- Adaptive management with decreased wood harvesting intensity.

Thus, we had two options for adaptive forest management (3 and 4) applying the 4C thinning algorithm for extracting higher (intensified) or lower (decreased) amounts of wood compared to BAU (wood harvest rate) from European forests. Thinning regimes (from above, below or middle) were assigned according to the tree species (e.g. from above for *Quercus* sp. and from below for *Picea abies*) based on current European standard practices (see references in S2-Table 1). Moreover, S2-Table 1 shows the percentage of stem biomass remaining in the stand after wood harvesting by thinning intervention.

S2-Table 1. Proportion of remaining stem biomass in the stand after thinning

Species	Thinning type	No management (%)	BAU (%)	Intensified thinning intensity (%)	Decreased thinning intensity (%)	Reference
Fagus sylvatica	Below	100	70	55	85	11,12,13,14,26,27
Picea abies	Below	100	70	55	85	1,6,7,8,9,10,19,20,21,22,23
Pinus sylvestris	Below	100	80	70	90	1,2,3,4,5,6,23
Quercus petraea	Above	100	85	77.5	92.5	2,14,15,16,17,19,24,25
Quercus robur	Above	100	85	77.5	92.5	2,14,16,17,19

We simulated forest growth, carbon budget, and responses to climate for the 21st century and a duration of 80 years, from 2011 to 2090 with the 4C model. During this period, we had three decision points at years 2020, 2030 and 2040. At these decision points, it was possible to change the management option, i.e. choosing one of the four options described above, and the option chosen at the last decision point continued through the end of the simulations (-**Fehler! Verweisquelle konnte nicht gefunden werden.**1).

S2-Figure 1. Management strategies. The management strategies were consisting of the decisions about No management (N), BAU, Intensified wood harvest (I) and Decreased wood harvest intensity (D) at three decision points 2020, 2030, and 2040. Therefore, 64 management options were simulated. For example, management option "DDI" applies Decrease, Decrease and Increase in wood harvesting intensity to periods 2020-2030, 2030-2040, and 2040-2090 respectively.

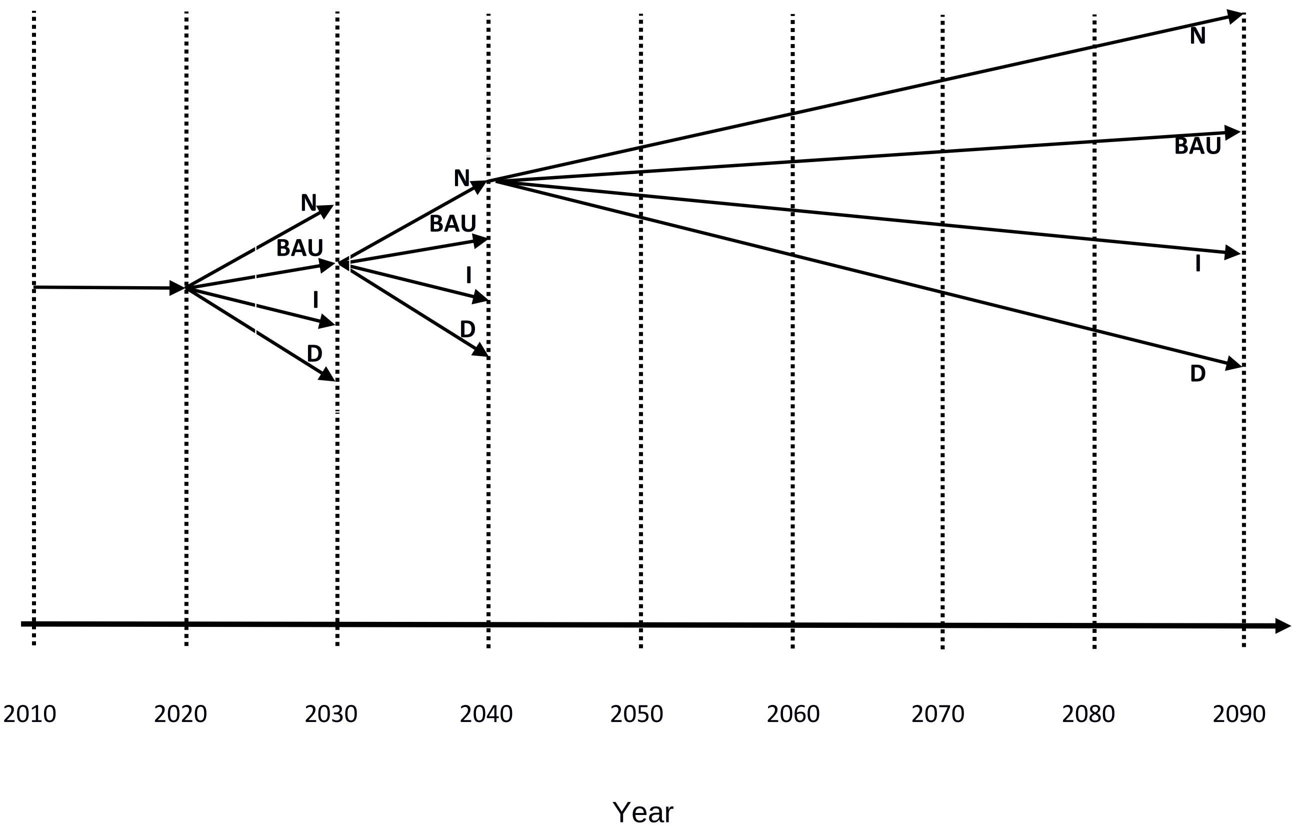

References

1. Pukkala, T. et al. A spatial yield model for optimizing the thinning regime of mixed stands of *Pinus sylvestris and Picea abies*. *Scandinavian Journal of Forest Research* **13,** 31-42 (1998).
2. Fürstenau, C. et al. Multiple-use forest management in consideration of climate change and the interests of stakeholder groups. *European Journal of Forest Research* **126,** 225-239 (2007).
3. González, J. et al. Optimising the management of *Pinus sylvestris* L. stand under risk of fire in Catalonia (north-east of Spain). *Annals of Forest Science* **62,** 493-501 (2005).
4. Lasch-Bonn, P. et al. Model-based analysis of management alternatives at stand and regional level in Brandenburg (Germany). *Forest Ecology and Management* **207,** 59-74 (2005).
5. Thren, M. Kiefernproduktionsprogramme. Erstellt auf der Basis von ertragskundlichen Versuchsflaechen und Einzelbaeumen. Diss. (Universität Freiburg, 1987).
6. Yrjölä, T. Forest management guidelines and practices in Finland, Sweden and Norway. Internal Report 11: 1–46 (European Forest Institute, 2002).
7. Hanewinkel, M. & Pretzsch, H. Modelling the conversion from even-aged to uneven-aged stands of Norway spruce (*Picea abies* L. Karst.) with a distance-dependent growth simulator. *Forest Ecology and Management* **134,** 55-70 (2000).
8. Abetz, P. Eine Entscheidungshilfe für die Durchforstung von Fichtenbeständen. *AFZ* 30, 666-667 (1975).
9. Sterba, H. Estimating potential density from thinning experiments and inventory data. *Forest Science* 33, 1022-1034 (1987).
10. Lähde, E. et al. Silvicultural alternatives in an uneven-sized forest dominated by *Picea abies*. *Journal of Forest Research* **15,** 14-20 (2010).
11. Schütz, J. P. Modelling the demographic sustainability of pure beech plenter forests in Eastern Germany. *Annals of Forest Science* **63,** 93-100 (2006).

12. Klädtke, J. Konzepte zur Buchen-Lichtwuchsdurchforstung. *AFZ-Der Wald* **56,**1047-1050 (2001).
13. Altherr, E. Wege zur Buchen-Starkholzproduktion. Bericht zur 15, 123-127. (Hauptversammlung des Baden-Württembergischen Forstvereins,1971).
14. Hein, S. & Dhôte, J. F. Effect of species composition, stand density and site index on the basal area increment of oak trees (*Quercus* sp.) in mixed stands with beech (*Fagus sylvatica* L.) in northern France. *Annals of Forest Science* **63,** 457-467 (2006).
15. Štefančík, I. Growth characteristics of oak (*Quercus petraea* [Mattusch.] Liebl.) stand under different thinning regimes. *Journal of Forest Science* **58,** 67-78 (2012).
16. Kerr, G. The effect of heavy or 'free growth' thinning on oak (*Quercus petraea* and *Q. robur*). *Forestry* **69,** 303-317 (1996).
17. Gutsch, M. et al. Management of mixed oak-pine forests under climate. *Forest Systems* **20,** 453-463 (2011).
18. Cutini, A. et al. Is anticipated seed cutting an effective option to accelerate transition to high forest in European beech (*Fagus sylvatica* L.) coppice stands? *Annals of Forest Science* **72,** 631-640 (2015).
19. Juodvalkis, A. et al. Effects of thinning on growth of six tree species in north-temperate forests of Lithuania. *European Journal of Forest Research* **124,** 187-192 (2005)..
20. Pape, R. Influence of thinning and tree diameter class on the development of basic density and annual ring width in *Picea abies*. *Scandinavian Journal of Forest Research* **14,** 27–37 (1999).
21. Wallentin, C. Thinning of Norway spruce. (SLU Reproenheten, Alnarp, 2007).
22. Mäkinen, H. & Isomäki, A. Thinning intensity and growth of Norway spruce stands in Finland. *Forestry* **77,** 349-364 (2004).
23. Bergh, J. et al. Long-term responses of Scots pine and Norway spruce stands in Sweden to repeated fertilization and thinning. *Forest Ecology and Management* **320,** 118-128 (2014).
24. Ningre, F. Comparison de différentes modalités d'éclaircie du chêne sessile. Premiers résultats d'un dispositif expérimental situé en forêt domaniale de Réno-Valdieu, (Orne). R.F.F. 17, 254-264 (1990).
25. Bréda, N, et al. (1995) Effects of thinning on soil and tree water relations, transpiration and growth in an oak forest (Quercus petraea (Matt.) Liebl.). *Tree Physiology* 15:295-306.
26. Cescatti, A. & Piutti, E. Silvicultural alternatives, competition regime and sensitivity to climate in a European beech forest. *Forest Ecology and Management* **102,** 213-223. (1998).
27. Mund, M. Carbon pools of European beech forests (Fagus sylvatica) under different silvicultural management. Forschungszentrum Waldökosysteme. Diss. (Georg-August-Universität Göttingen, 2004).
