# Supplementary material for: Realizing Mitigation Efficiency of European Commercial Forests by Climate Smart Forestry
Source: Sci Rep. 2018 Jan 10;8:345. doi: 10.1038/s41598-017-18778-w (PMC5762874; doi:10.1038/s41598-017-18778-w)
Supplement: Supplementary file 3 — Supplementary Info 3 [file 41598_2017_18778_MOESM3_ESM.pdf]

Title page

Supplementary 3

Title: Sensitivity analysis

To: Realizing Mitigation Efficiency of European Commercial Forests by Climate Smart Forestry

Authors: Rasoul Yousefpour[1]*, Andrey Lessa Derci Augustynczik[1], Christopher P.O. Reyer[2], Petra Lasch-Born[2], Felicitas Suckow[2], and Marc Hanewinkel[1]

[1] Chair of Forestry Economics and Forest Planning, Faculty of Environment and Natural Resources, University of Freiburg, Tennenbacherstr. 4, D-79106 Freiburg

[2] Potsdam Institute for Climate Impact Research (PIK), Telegraphenberg A62/1.05, D-14412 Potsdam

*Corresponding author (E-mail: rasoul.yousefpour@ife.uni-freiburg.de, Tel: +49-761-2033688, Fax: +49-761-2033690)

Sensitivity analysis

Interest rates and carbon sequestration

In order to evaluate the effect of the interest rate on carbon costs and mitigation (carbon sequestration) efficiency, besides the country-specific interest rate defined by the European Central Bank (see Supplementary 1), we computed carbon costs and sequestration potential applying a 0% and a 2% interest rate for each country and species. The results of the sensitivity analysis are shown below in S3-Figures 1, 2, and 3, respectively.

We observed that the sequestration potential when applying different interest rates remained unchanged for the maximum carbon sequestration policy scheme (G). As the management strategies yielding the maximum carbon sequestration are the same, regardless of the interest rate, there was no effect on the maximum sequestration potential (S3-Fig. 1a, 2a, and 3a). For the initial policy schemes A to D, there were stronger variations on the sequestration potential. In general, the sequestration potential increased with the lower interest rates. As we observed in S3-Figure 1a considering *Picea abies*, the sequestration potential for Sweden was within the class 400 to 700 PTE for policy A, for NPV maximization, whereas the same country presented sequestration potential of 100 to 400 PTE applying a 2% interest rate (S3-Fig. 3a). The overall increase in sequestration potential was 8.3% and 10.8% for the country-specific interest rate and for the 0% interest rate respectively, compared to the 2% interest rate. In addition, for the 2% interest rate we found that the sequestration increased substantially only when the preference for carbon sequestration was high, e.g. policies F and G.

The interest rates presented a strong impact on the carbon costs for all policy schemes (B to G). The carbon costs for all countries and species presented a strong increase from the 2% interest rate to the 0% interest rate (S3-Fig. 1b and 3b), with intermediary costs for the country-specific interest rates (S3-Fig. 2b). As it is possible to notice in S3-Figure 1b, for all species, except *Fagus sylvatica*, the carbon costs remained above 80.00 EUR/PTE for the majority of countries. Similarly, in S3-Figure 2b, with the country-specific interest rates, the majority of countries and species presented costs above 60.00 EUR/PTE, whereas the costs were below 60.00 EUR/PTE for the 2% interest rate (S3-Fig. 3 c). We perceived exceptions in Romania, Poland and Hungary, which presented lower costs compared to the 2% interest rate. In general, there was an increase of 108% on carbon costs considering a 0% interest rate and 42% considering the country-specific interest rates, in comparison with the 2% interest rate. For intermediary policies there were even stronger differences. For example, taking into account policy D, the costs in Scandinavian countries were five times higher; as this area was prioritized for carbon forestry under a 0% interest rate. Nevertheless, the costs remained below 60 EUR/PTE for all of the countries and species under the same policy, when the 2% and country-specific interest rates were applied.

The definition of target areas in our study was dependent on the carbon costs in each country, as well as the sequestration potential. Therefore, due to the impact of the interest rates on the carbon costs, we observed an effect on the allocation of climate-smart forestry. In S3-Figures 1c, 2c, and 3c there are substantial differences on the allocation of forests for carbon sequestration between the 0% and 2 % interest rates, as evidenced by the amount of the sequestration potential allocated in each country and species. For the 0% interest rate, there was an earlier allocation of forests to carbon sequestration, with high levels of carbon sequestration already for policy D, considering equal preference for carbon sequestration and NPV. Conversely, for the 2% interest rate and consequently more urgency for early harvesting revenues, forests remained under management focusing on timber production over a wider range of preferences for carbon sequestration and profitability. In this sense, high amounts of

carbon were sequestered mainly under policies F and G, with high preference for carbon sequestration. For the 0% interest rate, high amounts of carbon were sequestered already under policy B and C, due to a higher contribution of the remaining stock to forest profitability. Moreover, we observed that disregarding differences in economy of European countries changed the allocation of climate-smart forestry.

With the country-specific interest rate, and taking into account economic differences in local economy, climate-smart forestry was prioritized in eastern European countries, e.g. Poland, Romania, Slovakia, and Mediterranean areas for *Fagus sylvatica*. On the other hand, for the 2% and 0% interest rates, there was a greater contribution from Scandinavian countries, especially Sweden, and Germany. Moreover, for the 0% interest rates the share of *Picea abies* forests selected for carbon sequestration was higher compared to the country-specific interest rate.

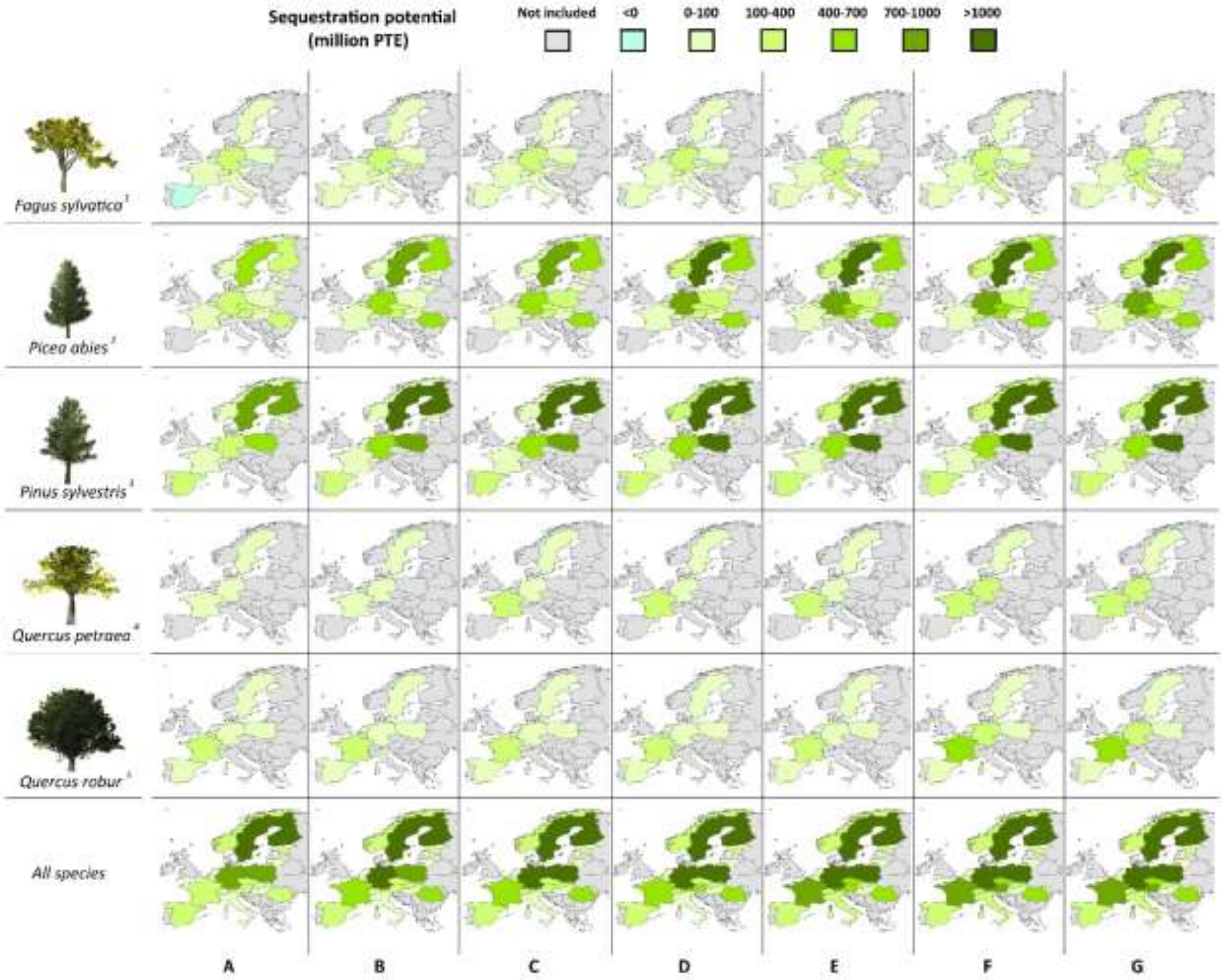

S3-Figure 1a. Carbon sequestration potential with increasing preference for carbon sequestration for a 0% interest rate. Figures S3 1-4 were created using the software QGIS Version 2.18.13 (http://www.qgis.org/en/site/) and paint.net version 4.0.12 (https://www.getpaint.net/). The base map was made with Natural Earth. Free vector and raster map data @ naturalearthdata.com and the tree images were retrieved from Pixabay database (1https://pixabay.com/pt/%C3%A1rvore-folhas-ramos-isolado-1638410/; 2https://pixabay.com/pt/pine-tree-pinheiro-%C3%A1rvore-natal-1480300/; 3https://pixabay.com/pt/%C3%A1rvore-sempre-viva-isolado-pinheiro-1702024/; 4https://pixabay.com/pt/%C3%A1rvore-folhas-ramos-isolado-1658801/; 5https://pixabay.com/pt/%C3%A1rvore-de-carvalho-%C3%A1rvore-1480225/).

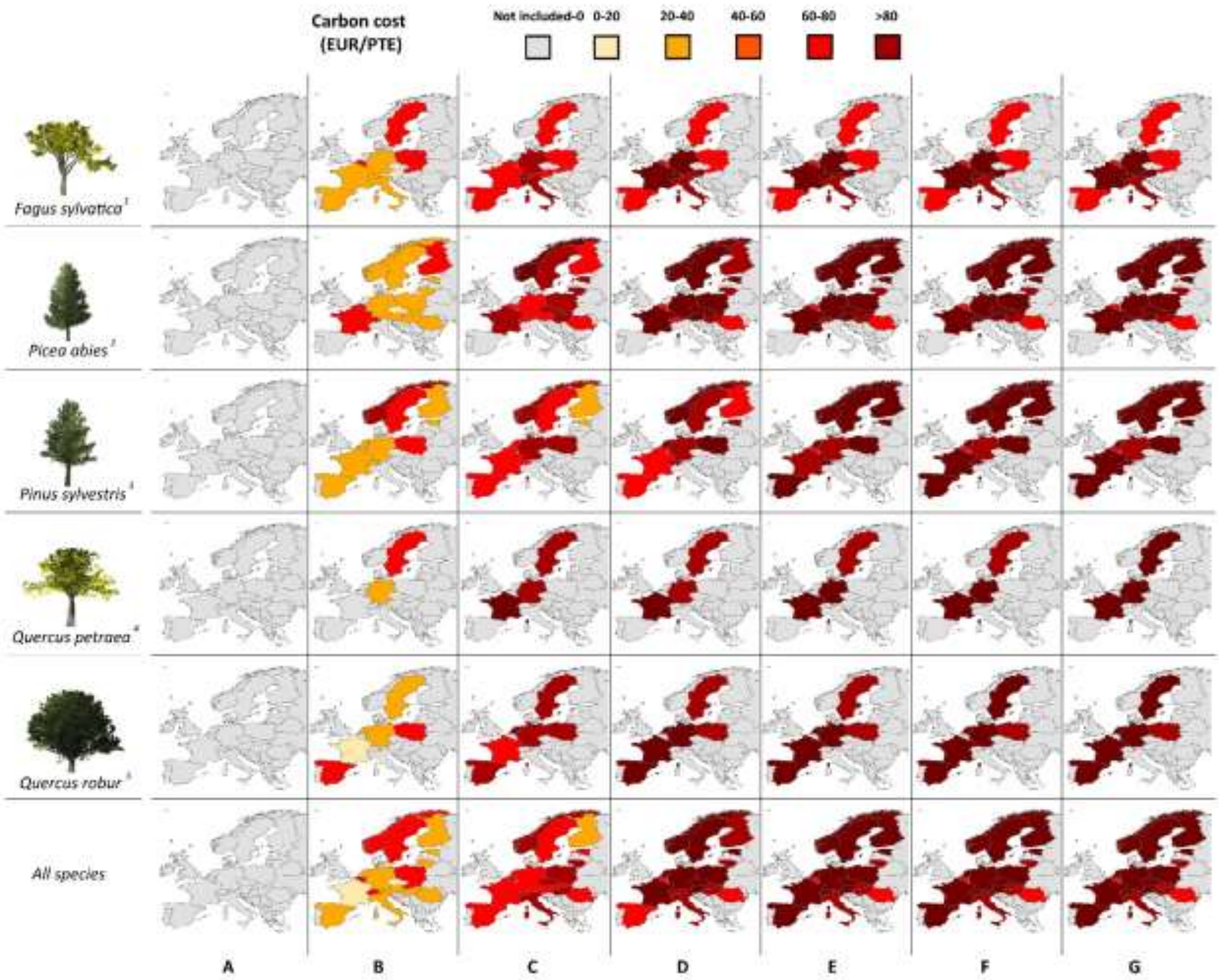

S3-Figure 1b. Carbon costs with increasing preference for carbon sequestration for a 0% interest rate. The figure was created using the software QGIS Version 2.18.13 (http://www.qgis.org/en/site/) and paint.net version 4.0.12 (https://www.getpaint.net/). The base map was made with Natural Earth. Free vector and raster map data @ naturalearthdata.com and the tree images were retrieved from Pixabay database (1https://pixabay.com/pt/%C3%A1rvore-folhas-ramos-isolado-1638410/; 2https://pixabay.com/pt/pine-tree-pinheiro-%C3%A1rvore-natal-1480300/; 3https://pixabay.com/pt/%C3%A1rvore-sempre-viva-isolado-pinheiro-1702024/; 4https://pixabay.com/pt/%C3%A1rvore-folhas-ramos-isolado-1658801/; 5https://pixabay.com/pt/%C3%A1rvore-de-carvalho-%C3%A1rvore-1480225/).

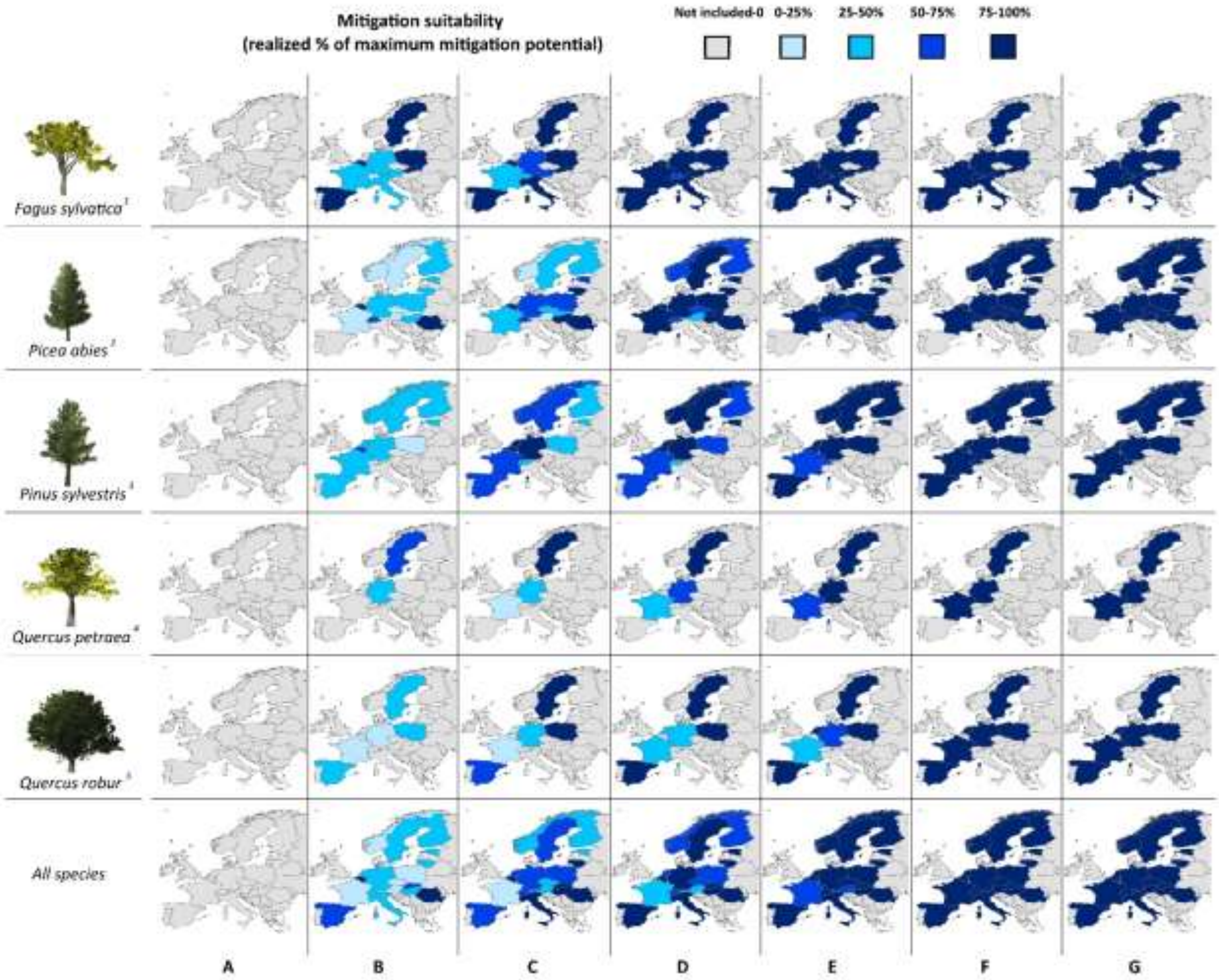

S3-Figure 1c. Mitigation suitability with increasing preference for carbon sequestration for a 0% interest rate. The figure was created using the software QGIS Version 2.18.13 (http://www.qgis.org/en/site/) and paint.net version 4.0.12 (https://www.getpaint.net/). The base map was made with Natural Earth. Free vector and raster map data @ naturalearthdata.com and the tree images were retrieved from Pixabay database (1https://pixabay.com/pt/%C3%A1rvore-folhas-ramos-isolado-1638410/; 2https://pixabay.com/pt/pine-tree-pinheiro-%C3%A1rvore-natal-1480300/; 3https://pixabay.com/pt/%C3%A1rvore-sempre-viva-isolado-pinheiro-1702024/; 4https://pixabay.com/pt/%C3%A1rvore-folhas-ramos-isolado-1658801/; 5https://pixabay.com/pt/%C3%A1rvore-de-carvalho-%C3%A1rvore-1480225/).

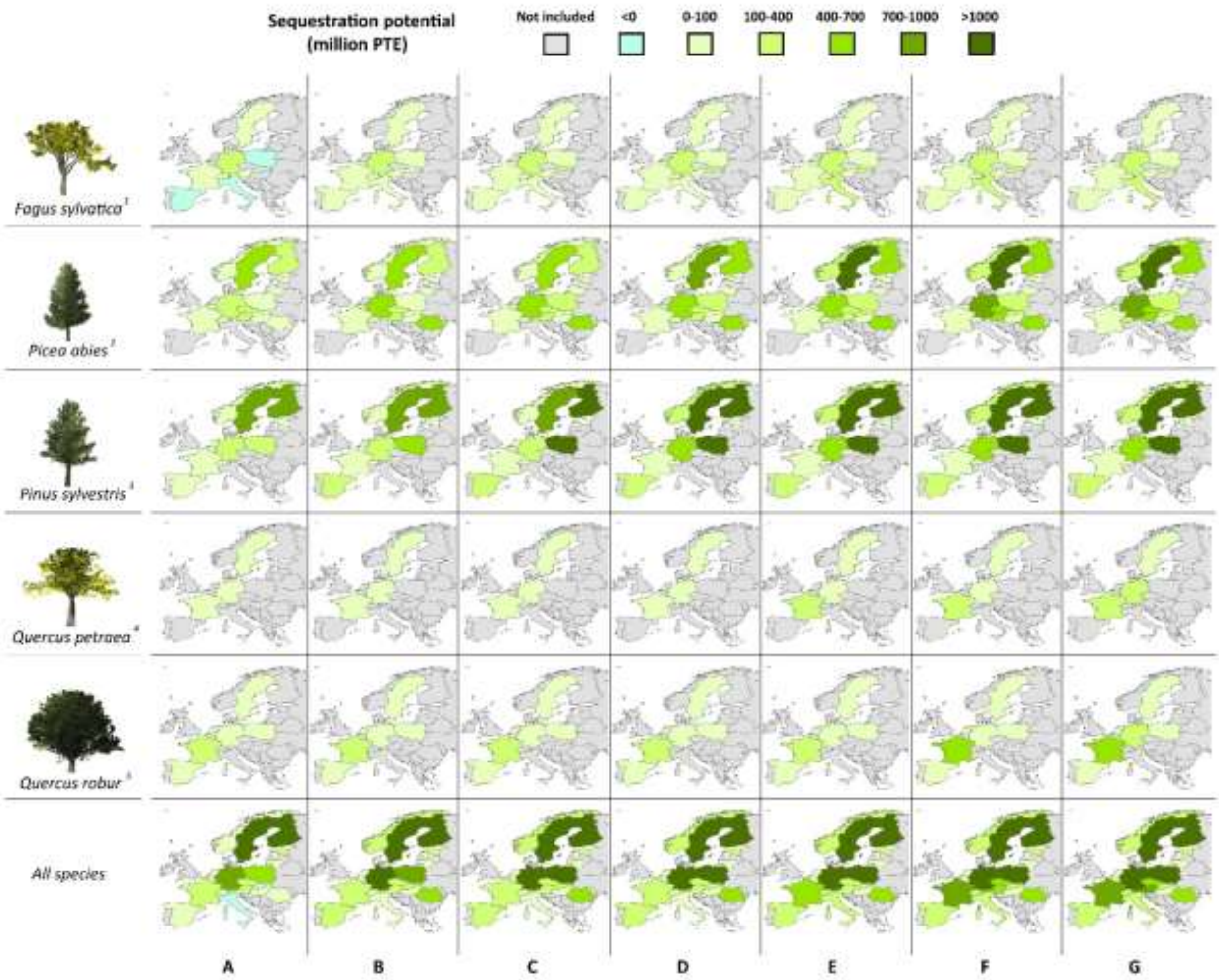

S3-Figure 2a. Carbon sequestration potential with increasing preference for carbon sequestration for a country-specific interest rate. The figure was created using the software QGIS Version 2.18.13 (http://www.qgis.org/en/site/) and paint.net version 4.0.12 (https://www.getpaint.net/). The base map was made with Natural Earth. Free vector and raster map data @ naturalearthdata.com and the tree images were retrieved from Pixabay database (1https://pixabay.com/pt/%C3%A1rvore-folhas-ramos-isolado-1638410/; 2https://pixabay.com/pt/pine-tree-pinheiro-%C3%A1rvore-natal-1480300/; 3https://pixabay.com/pt/%C3%A1rvore-sempre-viva-isolado-pinheiro-1702024/; 4https://pixabay.com/pt/%C3%A1rvore-folhas-ramos-isolado-1658801/; 5https://pixabay.com/pt/%C3%A1rvore-de-carvalho-%C3%A1rvore-1480225/).

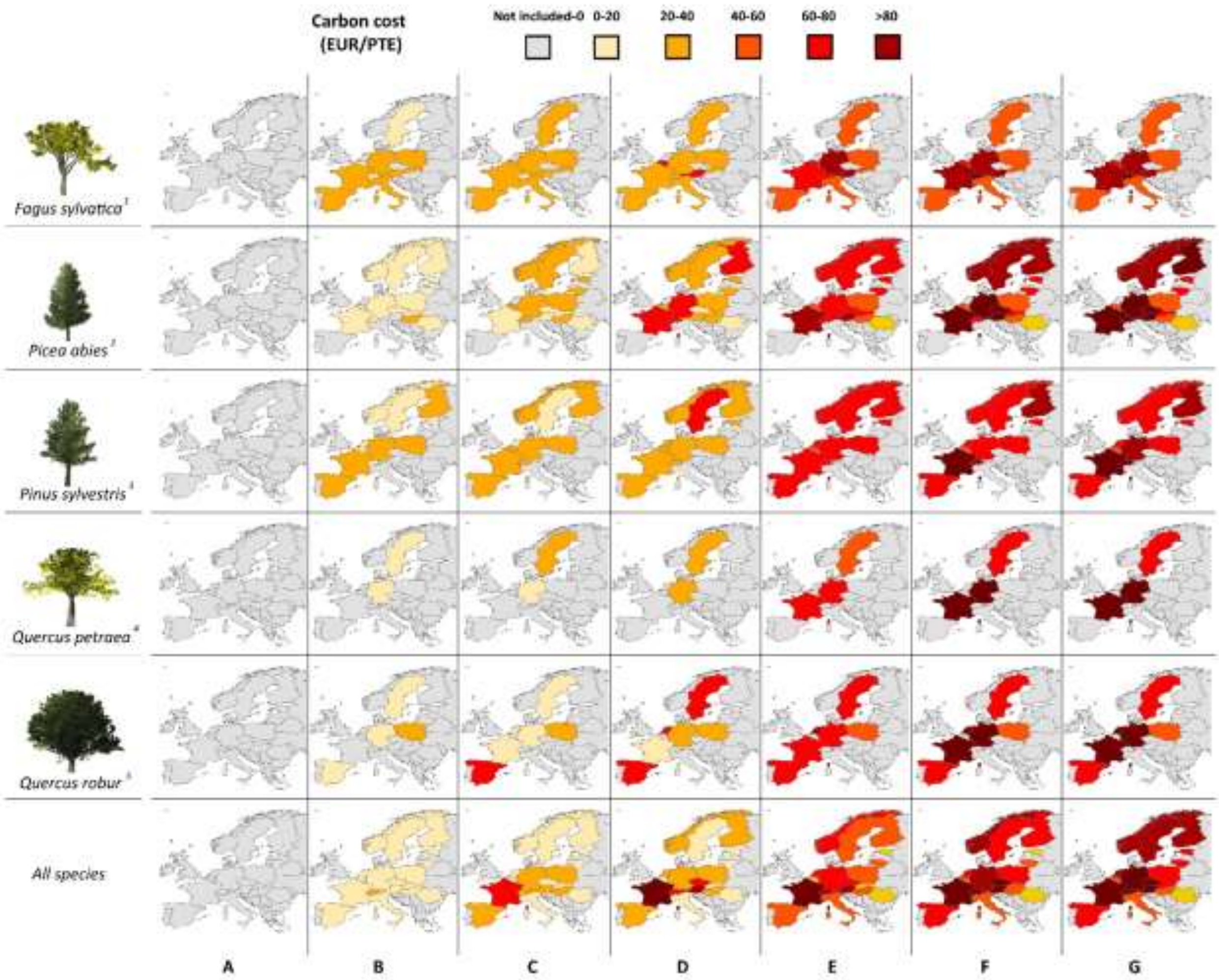

S3-Figure 2b. Carbon costs with increasing preference for carbon sequestration for a country-specific interest rate. The figure was created using the software QGIS Version 2.18.13 (http://www.qgis.org/en/site/) and paint.net version 4.0.12 (https://www.getpaint.net/). The base map was made with Natural Earth. Free vector and raster map data @ naturalearthdata.com and the tree images were retrieved from Pixabay database (1https://pixabay.com/pt/%C3%A1rvore-folhas-ramos-isolado-1638410/; 2https://pixabay.com/pt/pine-tree-pinheiro-%C3%A1rvore-natal-1480300/; 3https://pixabay.com/pt/%C3%A1rvore-sempre-viva-isolado-pinheiro-1702024/; 4https://pixabay.com/pt/%C3%A1rvore-folhas-ramos-isolado-1658801/; 5https://pixabay.com/pt/%C3%A1rvore-de-carvalho-%C3%A1rvore-1480225/).

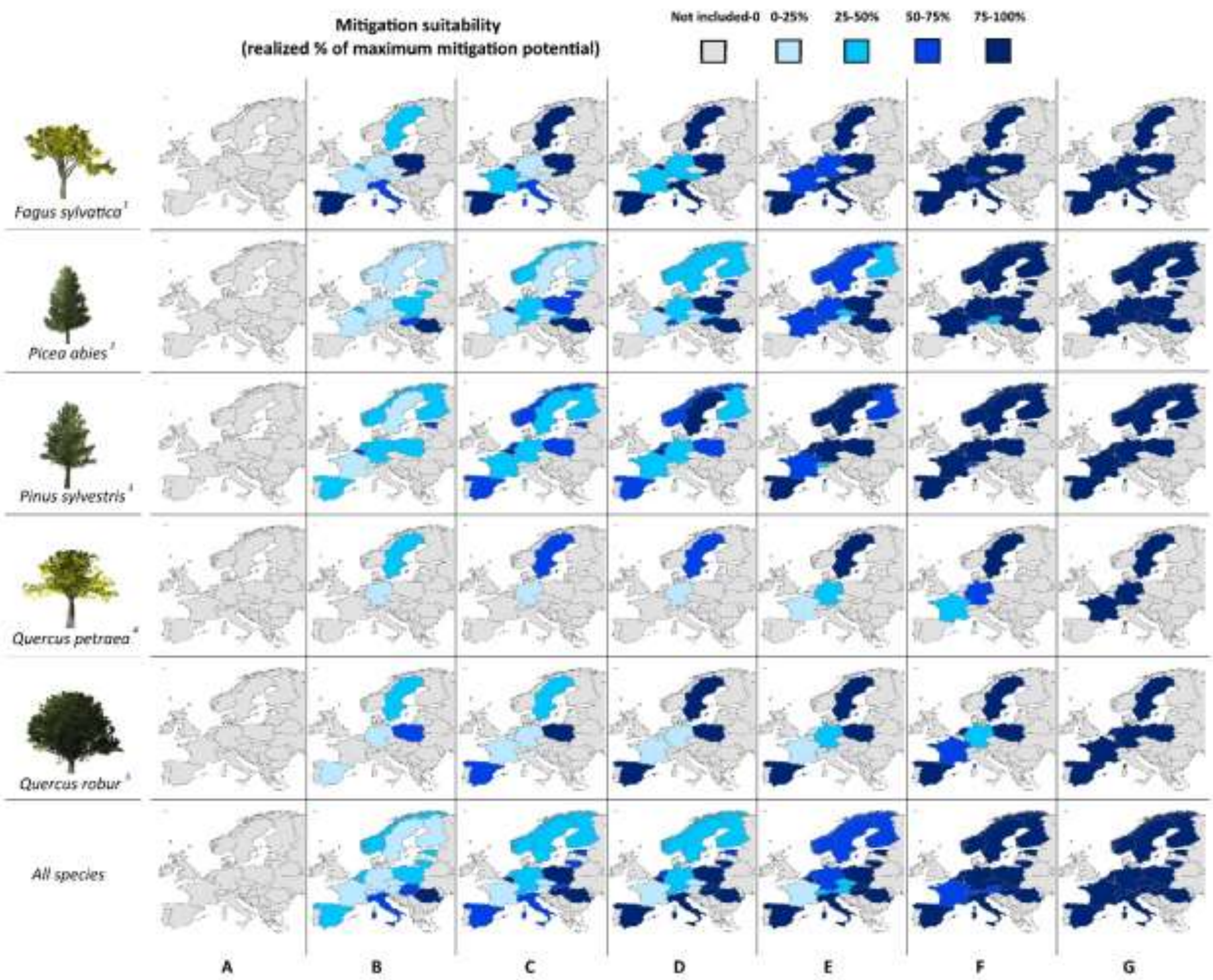

S3-Figure 2c. Mitigation suitability with increasing preference for carbon sequestration for a country-specific interest rate. The figure was created using the software QGIS Version 2.18.13 (http://www.qgis.org/en/site/) and paint.net version 4.0.12 (https://www.getpaint.net/). The base map was made with Natural Earth. Free vector and raster map data @ naturalearthdata.com and the tree images were retrieved from Pixabay database (1https://pixabay.com/pt/%C3%A1rvore-folhas-ramos-isolado-1638410/; 2https://pixabay.com/pt/pine-tree-pinheiro-%C3%A1rvore-natal-1480300/; 3https://pixabay.com/pt/%C3%A1rvore-sempre-viva-isolado-pinheiro-1702024/; 4https://pixabay.com/pt/%C3%A1rvore-folhas-ramos-isolado-1658801/; 5https://pixabay.com/pt/%C3%A1rvore-de-carvalho-%C3%A1rvore-1480225/).

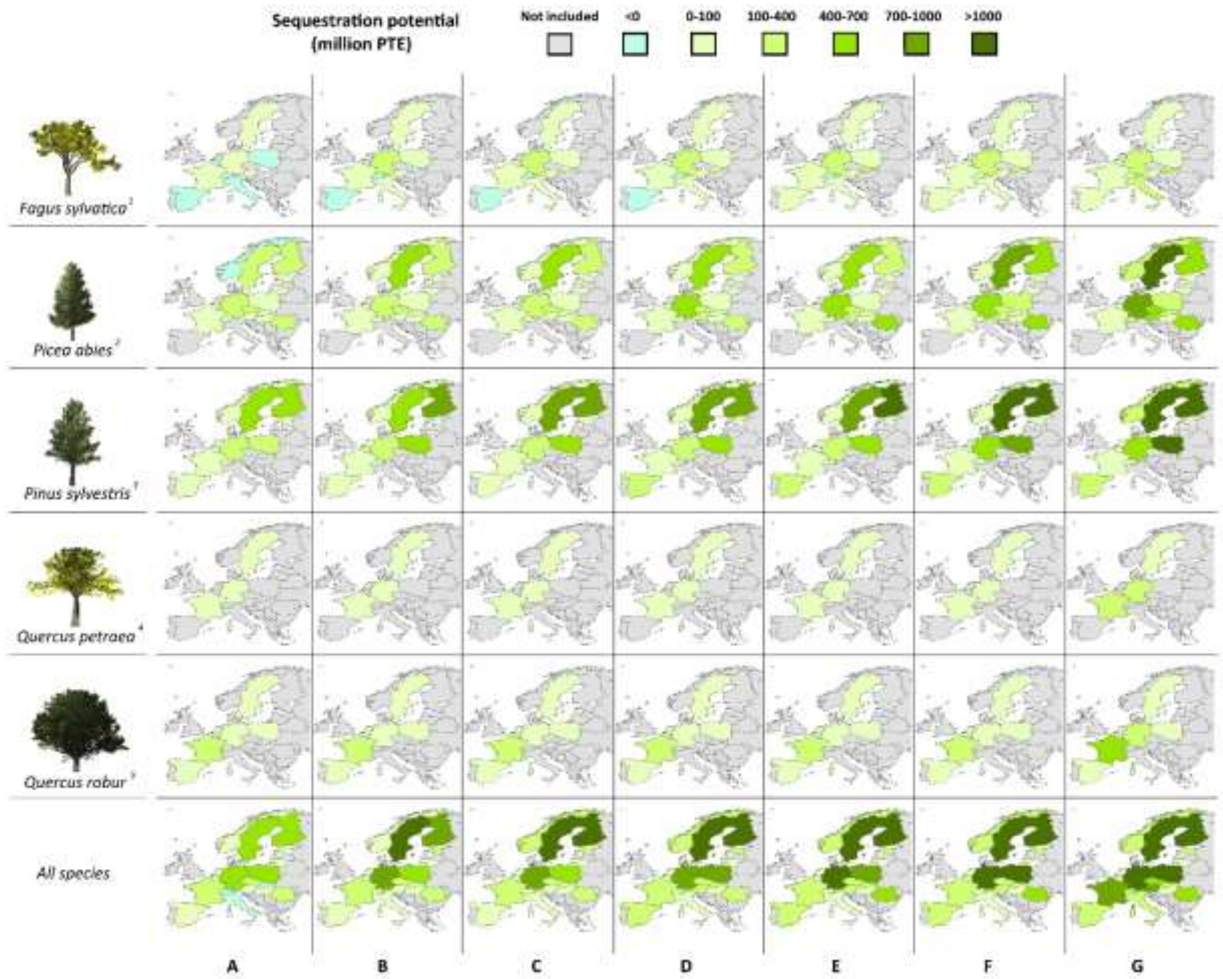

S3-Figure 3a. Carbon sequestration potential with increasing preference for carbon sequestration for a 2% interest rate. The figure was created using the software QGIS Version 2.18.13 (http://www.qgis.org/en/site/) and paint.net version 4.0.12 (https://www.getpaint.net/). The base map was made with Natural Earth. Free vector and raster map data @ naturalearthdata.com and the tree images were retrieved from Pixabay database (1https://pixabay.com/pt/%C3%A1rvore-folhas-ramos-isolado-1638410/; 2https://pixabay.com/pt/pine-tree-pinheiro-%C3%A1rvore-natal-1480300/; 3https://pixabay.com/pt/%C3%A1rvore-sempre-viva-isolado-pinheiro-1702024/; 4https://pixabay.com/pt/%C3%A1rvore-folhas-ramos-isolado-1658801/; 5https://pixabay.com/pt/%C3%A1rvore-de-carvalho-%C3%A1rvore-1480225/).

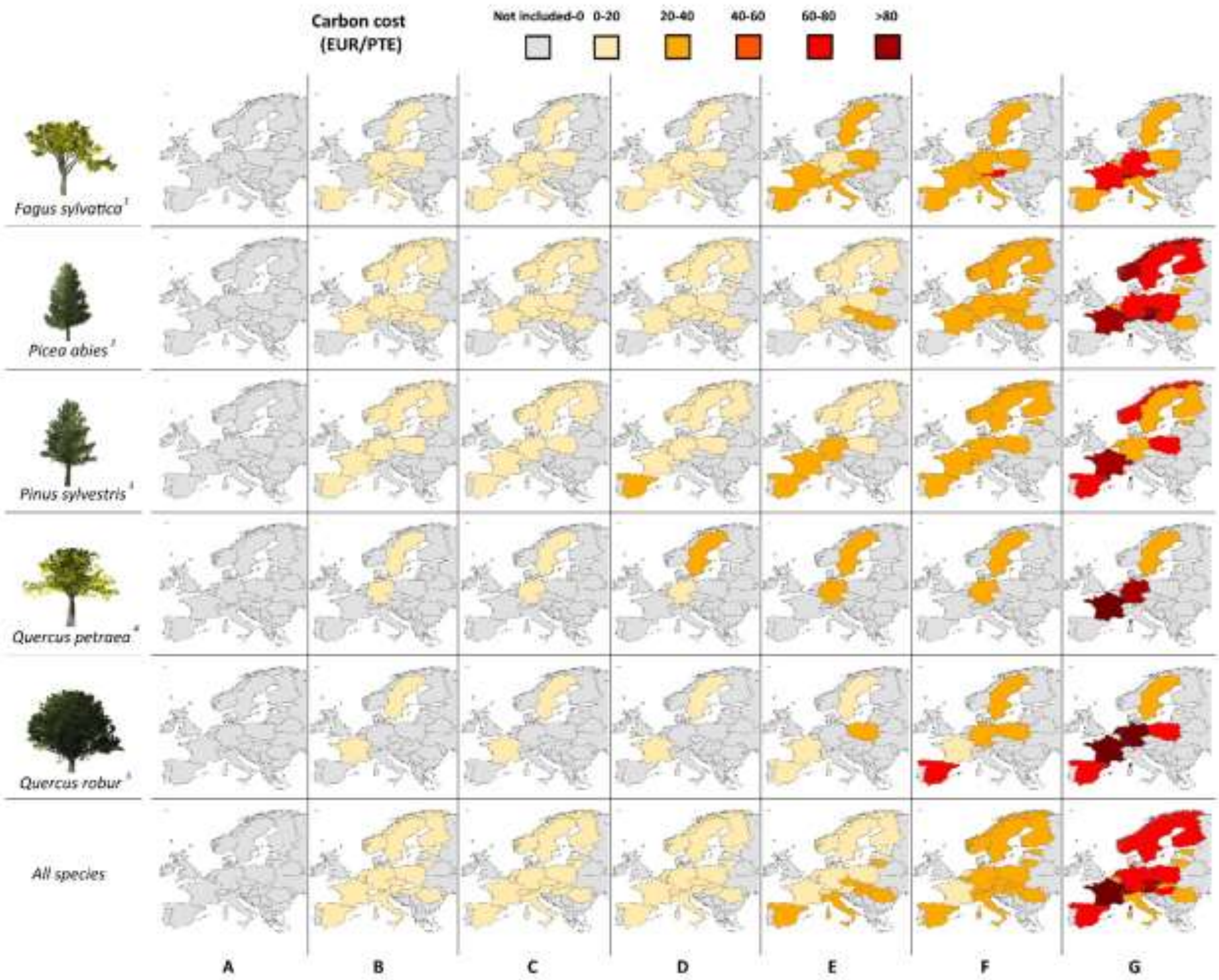

S3-Figure 3b. Carbon costs with increasing preference for carbon sequestration for a 2% interest rate. The figure was created using the software QGIS Version 2.18.13 (http://www.qgis.org/en/site/) and paint.net version 4.0.12 (https://www.getpaint.net/). The base map was made with Natural Earth. Free vector and raster map data @ naturalearthdata.com and the tree images were retrieved from Pixabay database (1https://pixabay.com/pt/%C3%A1rvore-folhas-ramos-isolado-1638410/; 2https://pixabay.com/pt/pine-tree-pinheiro-%C3%A1rvore-natal-1480300/; 3https://pixabay.com/pt/%C3%A1rvore-sempre-viva-isolado-pinheiro-1702024/; 4https://pixabay.com/pt/%C3%A1rvore-folhas-ramos-isolado-1658801/; 5https://pixabay.com/pt/%C3%A1rvore-de-carvalho-%C3%A1rvore-1480225/).

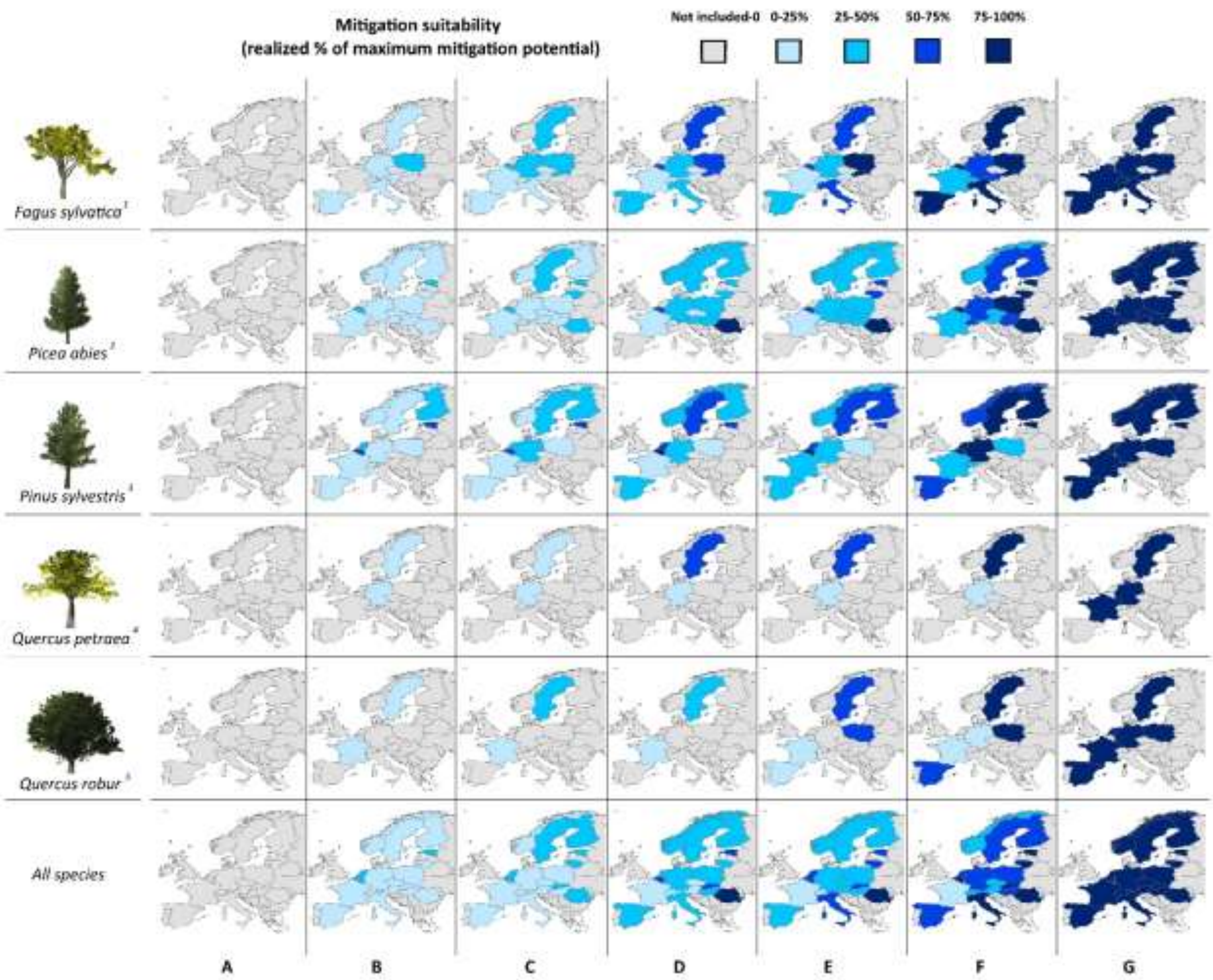

S3-Figure 3c. Mitigation suitability with increasing preference for carbon sequestration for a 2% interest rate. The figure was created using the software QGIS Version 2.18.13 (http://www.qgis.org/en/site/) and paint.net version 4.0.12 (https://www.getpaint.net/). The base map was made with Natural Earth. Free vector and raster map data @ naturalearthdata.com and the tree images were retrieved from Pixabay database (1https://pixabay.com/pt/%C3%A1rvore-folhas-ramos-isolado-1638410/; 2https://pixabay.com/pt/pine-tree-pinheiro-%C3%A1rvore-natal-1480300/; 3https://pixabay.com/pt/%C3%A1rvore-sempre-viva-isolado-pinheiro-1702024/; 4https://pixabay.com/pt/%C3%A1rvore-folhas-ramos-isolado-1658801/; 5https://pixabay.com/pt/%C3%A1rvore-de-carvalho-%C3%A1rvore-1480225/).

Carbon discounting

In order to evaluate the effect of applying a time preference for carbon sequestration, we performed the analysis of carbon costs and sequestration potential considering no carbon discounting and the country-specific interest rate for computing forest NPV. S3-Figure 4a, 4b and 4c present the sequestration potential, carbon costs and suitability of countries and species for carbon sequestration and a climate-smart forestry under this framework. As displayed in S3-Figure 4a, the sequestration potential increased significantly compared to S3-Figure 2a, applying time preference for carbon sequestration. When carbon was discounted, the amount of sequestration at the end of the 80-years simulation period had a contribution nearly five times smaller than the carbon sequestered without time preference. Therefore, the sequestration potential increased with no time preference for carbon. For example, the sequestration of *Pinus sylvestris* under policy scheme A (max NPV) remained in the class from 700-1000 Million PTE when the carbon was discounted, whereas it remained above 1000 PTE with no time preference for carbon. Consequently, with an equal NPV and higher carbon sequestration levels the carbon costs reduced dramatically when no time preference was applied. This behavior was observed for all policy schemes, as illustrated in S3-Figure 7b.

Although the sequestration potential and carbon costs varied significantly when the time preference for carbon was removed, the efficient areas for climate-smart forestry remained essentially unchanged (S3-Fig. 7c). *Picea abies* in eastern European countries and *Pinus sylvestris* in Poland and Scandinavia remained as the most efficient areas for climate-smart forestry. In addition, we observed an increased contribution of *Picea abies* in Germany and Norway under this framework.

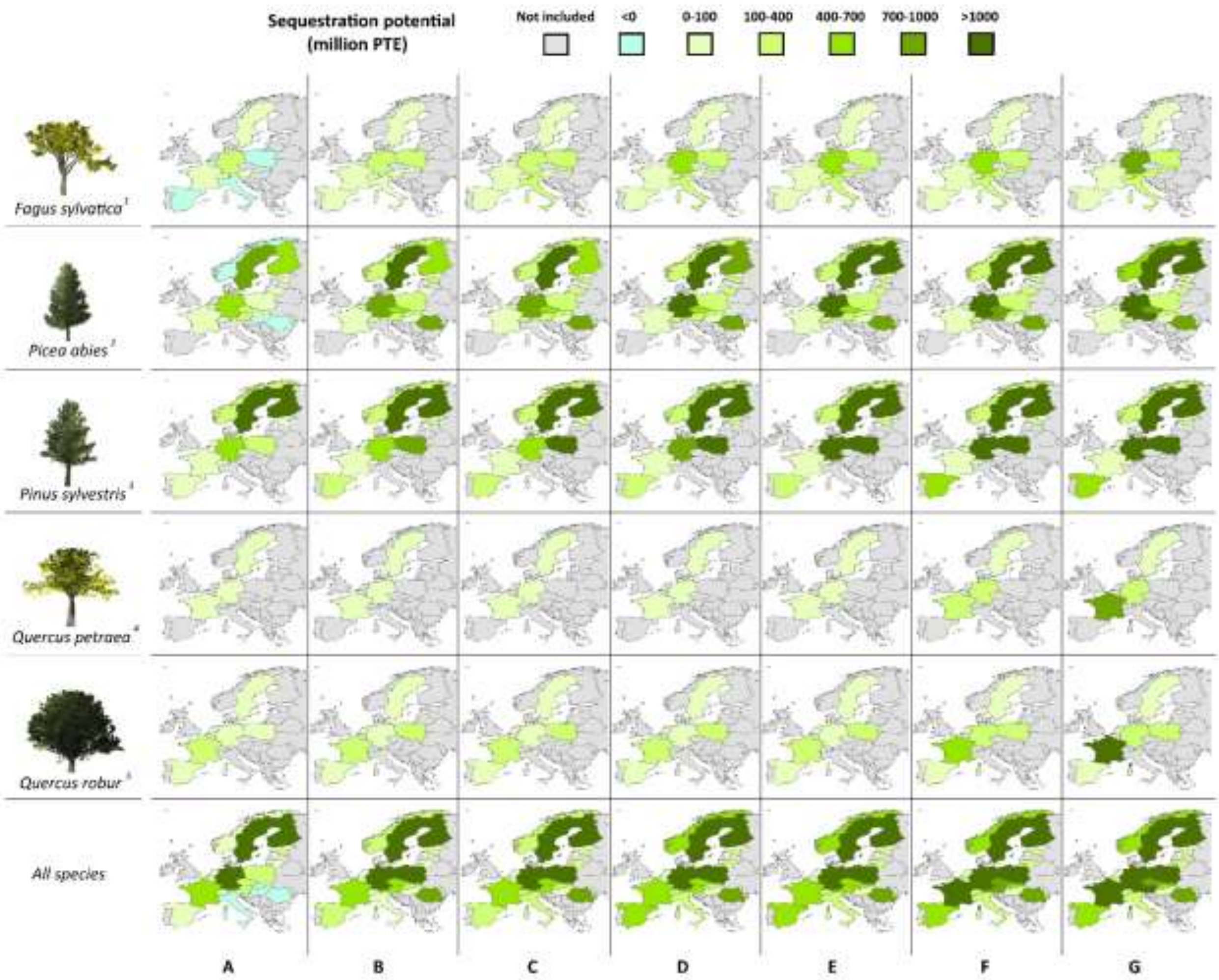

S3-Figure 4a. Carbon sequestration potential with increasing preference for carbon sequestration for a country-specific interest rate and no time preference for carbon sequestration. The figure was created using the software QGIS Version 2.18.13 (http://www.qgis.org/en/site/) and paint.net version 4.0.12 (https://www.getpaint.net/). The base map was made with Natural Earth. Free vector and raster map data @ naturalearthdata.com and the tree images were retrieved from Pixabay database (1https://pixabay.com/pt/%C3%A1rvore-folhas-ramos-isolado-1638410/; 2https://pixabay.com/pt/pine-tree-pinheiro-%C3%A1rvore-natal-1480300/; 3https://pixabay.com/pt/%C3%A1rvore-sempre-viva-isolado-pinheiro-1702024/; 4https://pixabay.com/pt/%C3%A1rvore-folhas-ramos-isolado-1658801/; 5https://pixabay.com/pt/%C3%A1rvore-de-carvalho-%C3%A1rvore-1480225/).

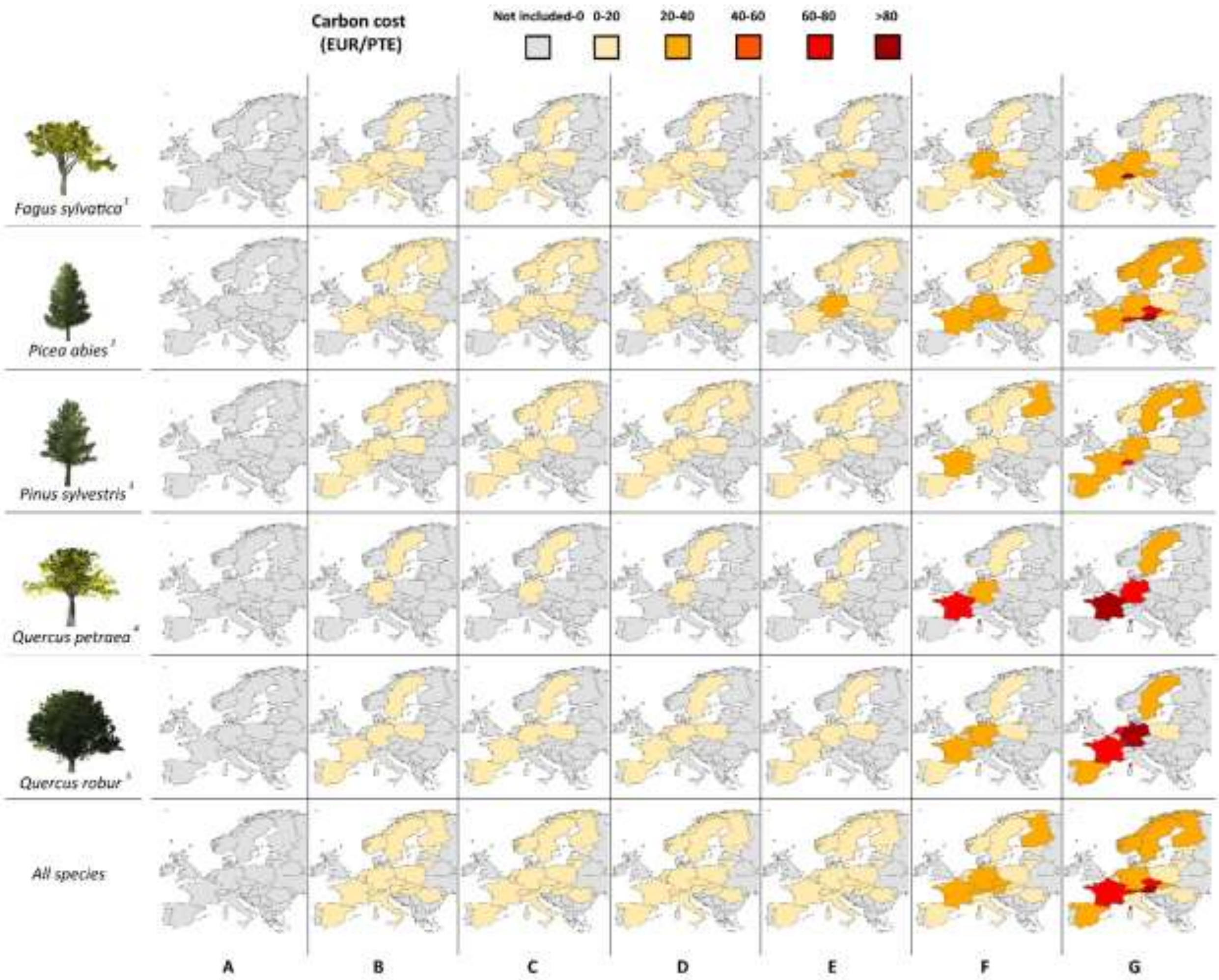

S3-Figure 4b. Carbon costs with increasing preference for carbon sequestration for a country-specific interest rate and no time preference for carbon sequestration. The figure was created using the software QGIS Version 2.18.13 (http://www.qgis.org/en/site/) and paint.net version 4.0.12 (https://www.getpaint.net/). The base map was made with Natural Earth. Free vector and raster map data @ naturalearthdata.com and the tree images were retrieved from Pixabay database (1https://pixabay.com/pt/%C3%A1rvore-folhas-ramos-isolado-1638410/; 2https://pixabay.com/pt/pine-tree-pinheiro-%C3%A1rvore-natal-1480300/; 3https://pixabay.com/pt/%C3%A1rvore-sempre-viva-isolado-pinheiro-1702024/; 4https://pixabay.com/pt/%C3%A1rvore-folhas-ramos-isolado-1658801/; 5https://pixabay.com/pt/%C3%A1rvore-de-carvalho-%C3%A1rvore-1480225/).

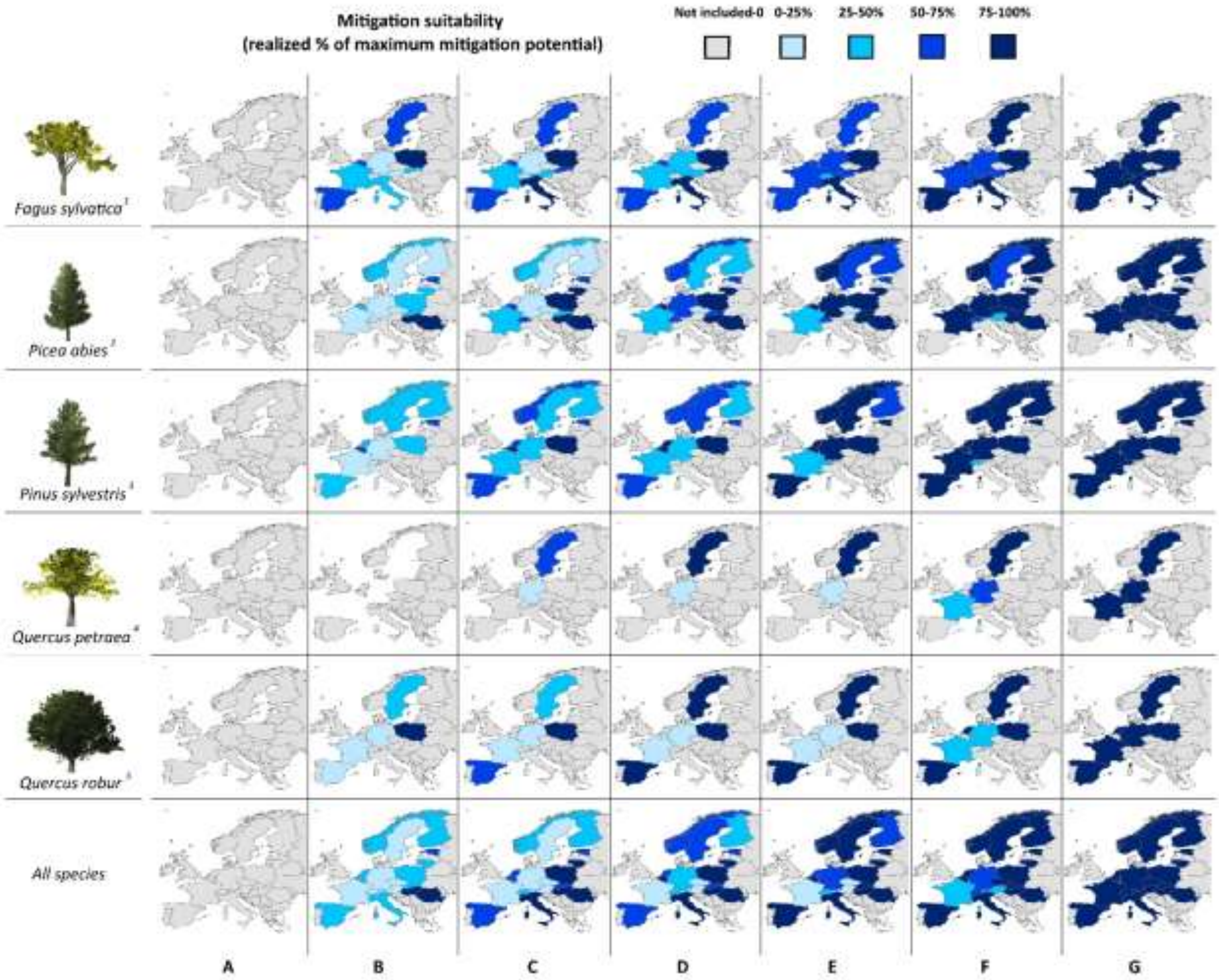

S3-Figure 4c. Mitigation suitability with increasing preference for carbon sequestration for a country-specific interest rate and no time preference for carbon sequestration. The figure was created using the software QGIS Version 2.18.13 (http://www.qgis.org/en/site/) and paint.net version 4.0.12 (https://www.getpaint.net/). The base map was made with Natural Earth. Free vector and raster map data @ naturalearthdata.com and the tree images were retrieved from Pixabay database (1https://pixabay.com/pt/%C3%A1rvore-folhas-ramos-isolado-1638410/; 2https://pixabay.com/pt/pine-tree-pinheiro-%C3%A1rvore-natal-1480300/; 3https://pixabay.com/pt/%C3%A1rvore-sempre-viva-isolado-pinheiro-1702024/; 4https://pixabay.com/pt/%C3%A1rvore-folhas-ramos-isolado-1658801/; 5https://pixabay.com/pt/%C3%A1rvore-de-carvalho-%C3%A1rvore-1480225/).

The time preference for carbon sequestration plays an important role in its economic costs. We analyzed the impacts of applying a time preference (2% discount rate) and no time preference (0% discount rate) for carbon sequestration on the supply curve, marginal and total costs of carbon (S3 Fig. 5). There were significant effects on both carbon costs and sequestration potential. When the carbon sequestration was not discounted (timely indifferent realization of mitigation), the sequestration potential increased drastically, surpassing 20 Billion PTE. Simultaneously, we observed a strong decrease in carbon costs. For example, the NPV reduction from the max NPV (policy A) to the max carbon sequestration (policy G) remained constant with or without time preference for carbon sequestration. Nevertheless, applying no time preference for carbon sequestration caused an increase in sequestration potential, resulting in lower opportunity costs per unit of carbon sequestered. This phenomenon caused the reduction in the marginal and total costs. Marginal costs remained below 5.00 EUR/PTE with no time preference for carbon sequestration (6e), whereas it surpassed 10.00 EUR/PTE when carbon was discounted (6b). Comparing figures 6c and 6f, at an equal cost level, the sequestration potential was substantially higher in figure 6f, without time preference for carbon. Disregarding discounting for carbon sequestration, up to 26 billion tons of carbon can be sequestered in our simulation framework and on average for all climate change scenarios. Similarly, all figures are higher comparing with 2% rate whenever the time preference for carbon sequestration is ignored. The highest accumulation of carbon in European forests may be realized using HAD A1B scenario (32 billion tons of carbon). Applying a 2% discount rate for implementing an urgent mitigation potential (time preference), realizes a lower PTE carbon with almost the same level of carbon costs.

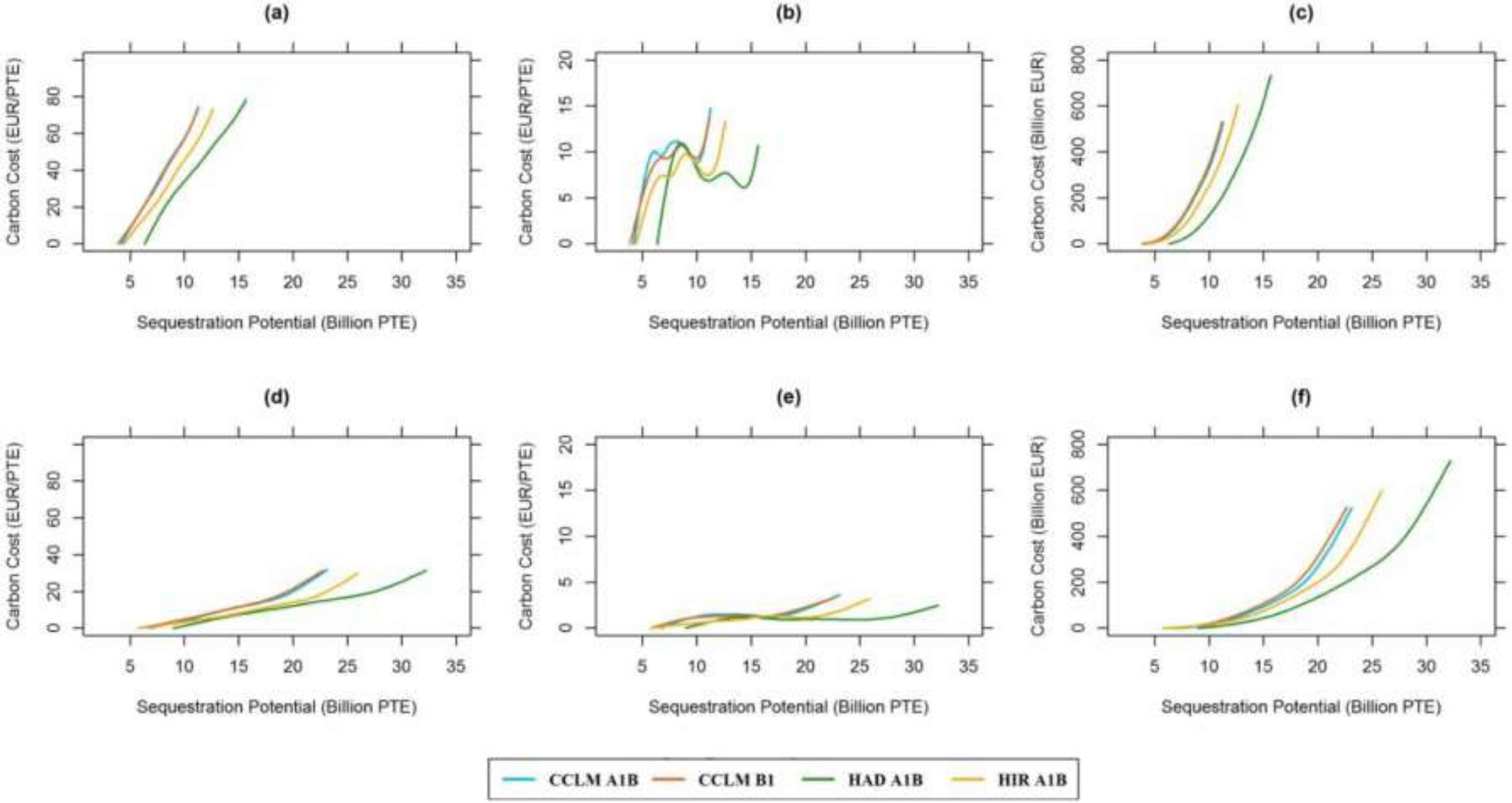

S3-Figure 5. Sensitivity analysis on time preference for carbon sequestration. The figure shows the effect of discounting carbon sequestration on the supply, marginal, and total costs for increasing the carbon sequestration potentials in Europe, with (a), (b) and (c) showing the marginal costs for the country-specific interest rate and a 2% carbon discounting. Similarly, (d), (e), and (f) show the supply, marginal, and total cost for no time preference for carbon sequestration (0% discounting).

Discussions on the sensitivity analysis

The interest rate presented important impacts on the sequestration potential for policy schemes with greater weight for the NPV maximization and major influence on carbon costs for all policy schemes. Considering the NPV maximization (policy A), when the growth rate of forests become smaller than the interest rate applied, it is more interesting from the economic point of view to harvest at the earliest point possible. Conversely, with a decreasing interest rate, the contribution of the revenues generated by the wood harvested at the later periods of the simulation gain importance and management strategies maintaining higher stock become more profitable. Thus, for the 0% interest rate with a discount factor equal to one over the simulation period, the management strategy yielding the maximum NPV was the management strategy that maximized the harvested wood volume. As management strategies maximizing the wood volume presented lower harvesting intensity than the management maximizing the NPV for the 2% interest rate, which promoted early harvestings in order to anticipate revenues, the carbon sequestration generated by the maximum NPV management increased for the 0% interest rate. The initial policy schemes presented higher carbon sequestration for 0% compared to the country-specific and 2% interest rates. On the other hand, as the maximum carbon sequestration management remains unchanged, management strategies with higher priority for carbon sequestration presented similar sequestration potential for all interest rates.

The carbon costs in our study were sensitive to the interest rates applied, once they were computed based on the management strategy yielding the maximum NPV. Accordingly, due to the direct impacts of the interest rate on the NPV, the costs varied among the interest rates applied, with increasing carbon costs as interest rates decreased. When the interest rate reduced, the profitability of forest harvesting increased, augmenting the opportunity costs for reducing management intensity aiming at increasing carbon sequestration. In addition, with the application of the 0% and the country-specific interest rates, (with the majority of countries applying interest rates below 2%), the discount factor of the harvesting revenue decreased, while the absolute difference between the NPV yielded by the maximum NPV management and the maximum carbon management increased. Simultaneously, the difference between the carbon sequestered by the maximum NPV management and by the maximum carbon sequestration management was reduced. Consequently, a higher cost for sequestering a smaller amount of carbon occurred for the 0% interest rate, compared to the 2% interest rate. In contrast, for Hungary, Poland and Romania, the carbon costs were reduced when the country-specific interest rates were applied. This behavior arose from interest rates higher than 2% practiced in these countries (see Supplementary 1). Our results corroborate with the Adams et al.[1] evaluation of the costs of increasing carbon sequestration through forest management, and changes in the forest area resulted from the variation of the welfare of forest and agriculture markets with the inclusion of sequestration targets. The results showed a decrease in average carbon costs from $35.00/mt to $26.00/mt with the increase in the interest rate from 3 to 5%. Anthoff et al.[2] and Greenstone et al. [3] found similar results studying social carbon costs, in order to define the benefits of reducing carbon emissions and reported decreasing social carbon costs with increasing interest rates.

Considering the impact of the interest rates on the carbon costs, the allocation of countries and species to mitigation policies was affected. For the 0% interest rate, areas were designated fully to carbon sequestration at lower preferences for carbon, compared to the other interest rates applied. This behavior arises from the higher contribution of standing stock to NPV under this interest rate, leading to higher profitability of lower wood utilization, reducing the incompatibility of both objectives. Conversely, for the 2% interest rate, areas were designated for carbon sequestration only with high preferences for carbon, as there were substantial NPV losses with decreased wood utilization. Therefore, it is very necessary to anticipate revenues from selling wood products. Moreover, oak species were designated for

carbon sequestration only at high levels of preference for carbon, due to the high profitability of these forests, a result of the high wood prices and reasonable harvesting costs.

We found for the country-specific interest rate, that carbon sequestration was allocated initially to countries with lower forest profitability, resulting from higher interest rates (e.g. Poland) and species with lower wood prices (e.g. *Fagus sylvatica*). The inclusion of more expensive areas gradually increased when the preference for carbon increased. On the other hand, when regional economic differences were disregarded (applying unique 0% and 2% interest rates), the contribution of Scandinavian countries and Germany increased. These areas present high sequestration potential, and without differentiation in the discounting schemes they became more interesting for climate-smart forestry. These countries present interest rates lower than in eastern European countries, therefore with the application of an equal interest rate they became relatively cheaper. In this sense, it was possible to apply management strategies with decreased harvesting intensity instead of setting these areas aside, allowing the achievement of high sequestration potentials with reasonable costs.

The effect of applying time preference for carbon sequestration affected the costs significantly. However, the allocation of climate-smart forestry remained essentially unchanged. We perceived an increased contribution to carbon sequestration of *Picea abies* in Norway and Germany. This behavior likely arose from an intensified carbon sequestration at the end of simulation period for these countries, which was weakened by the discounting factor when time preferences were applied, thus increasing the efficiency of these areas for climate-smart forestry.

Carbon sequestration policies and management interventions

S3-Figure 10 presents the relative frequency of the four management interventions considered in the simulation (Forest conservation (no management), BAU, Intensified harvesting and Decreased harvesting), for all countries and climate change scenarios, according to species and policy scheme.

For the maximum carbon sequestration (policy G), the results obtained were identical regardless of the interest rate applied. For this policy, nearly all management interventions consisted of forest conservation (no thinning interventions), indicating that a no intervention policy scheme is to be adopted if the manager’s objective is solely to maximize the carbon amount *in situ*. We observed exceptions for *Picea abies* and *Pinus sylvestris* in specific cases, in which no management strategy, consisting only of no thinning interventions, was not selected. Nevertheless, the proportion of other management interventions remained below 1% for both species applying policy G.

Similarly, for policies with higher preference for carbon sequestration, management interventions were similar considering the different interest rates applied (S3-Fig. 10a, 10b and 10c). Applying policy F, no thinning interventions were predominant, however it did include mixed management interventions when the 2% interest rate was applied, especially for the oak species. For the 0% interest rate, the proportion of decreased harvesting was higher compared to the 2% and country-specific interest rates. For all interest rates, oak species presented higher harvesting intensity under carbon sequestration preference, compared to *Fagus sylvatica* and the conifers. With increasing preference for NPV (Policies A to C) and equal preference (policy D), it was necessary to diversify the management interventions.

For policy D, no thinning and the decreased wood harvesting intensity was selected under the efficient carbon sequestration policy, indicating that in order to increase carbon sequestration in European forests it would be necessary to decrease harvesting intensity in the future. Overall, for the country-specific interest rates, the decreased wood harvesting

intervention and no thinning management strategies presented a frequency above 40% and the remaining interventions presented a frequency below 10%, except for where oak species occur that necessitated a balanced mix of management strategies. For the 2% interest rate there was an increase in the frequency of decreased wood harvesting and a concurrent reduction on the proportion of no thinning, compared to the 0% and country-specific interest rates. In addition, under this policy dissimilarities appeared regarding different species. *Fagus sylvatica, Picea abies* and *Pinus sylvestris* presented high frequency of decreased wood harvesting interventions, whereas both oak species showed a substantially higher percentage of increased wood harvesting for the 2% interest rate.

For policies A to C (with preference for NPV), increased wood harvesting and BAU interventions were predominant. With the higher interest rate of 2% (S3-Fig. 10c), the proportion of intensified harvesting was largest, especially for oak species. On the other hand, for the 0% and country-specific interest rate, a diversified set of interventions occurred, as the contribution of standing volume at the end of the simulation period increased.

Comparing S3-Figures 10b and 10d, we observe the effect of applying a time preference for carbon sequestration. Management interventions were similar with higher preference for NPV (A to C), presenting diversified management actions with a predominance of increased harvesting. For policies D to F, with increasing preference for carbon sequestration, a higher harvesting intensity for oaks was observed in S3-Figure 10d, with no time preference for carbon sequestration. In general, the amount of no thinning interventions was slightly higher when the time preference for carbon was applied. For policy G (max carbon sequestration) the management was identical, with nearly 100% of forests needing no thinning interventions.

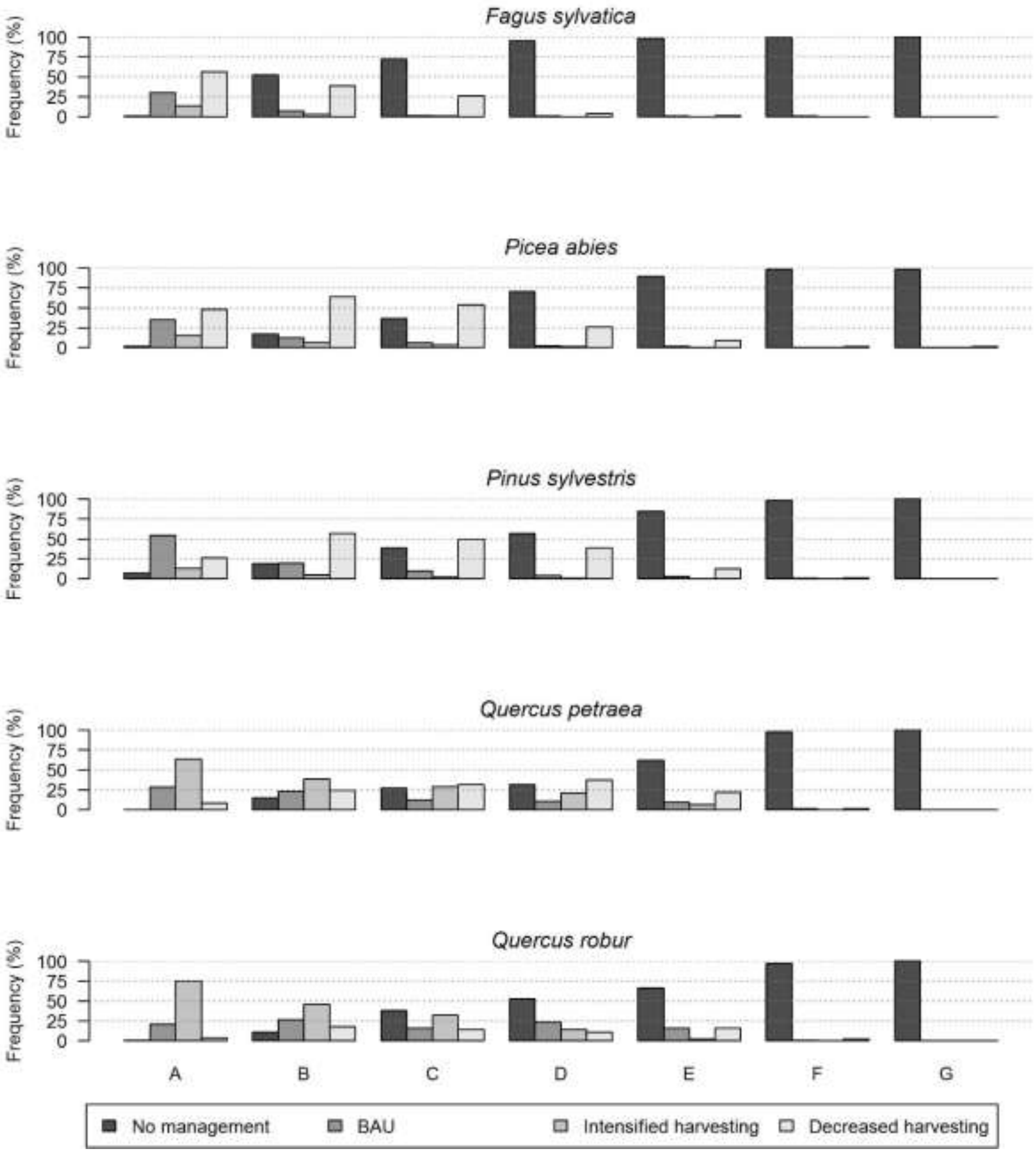

S3-Figure 6a. Relative frequency of each management intervention with increasing preference for carbon sequestration, considering a 0% interest rate.

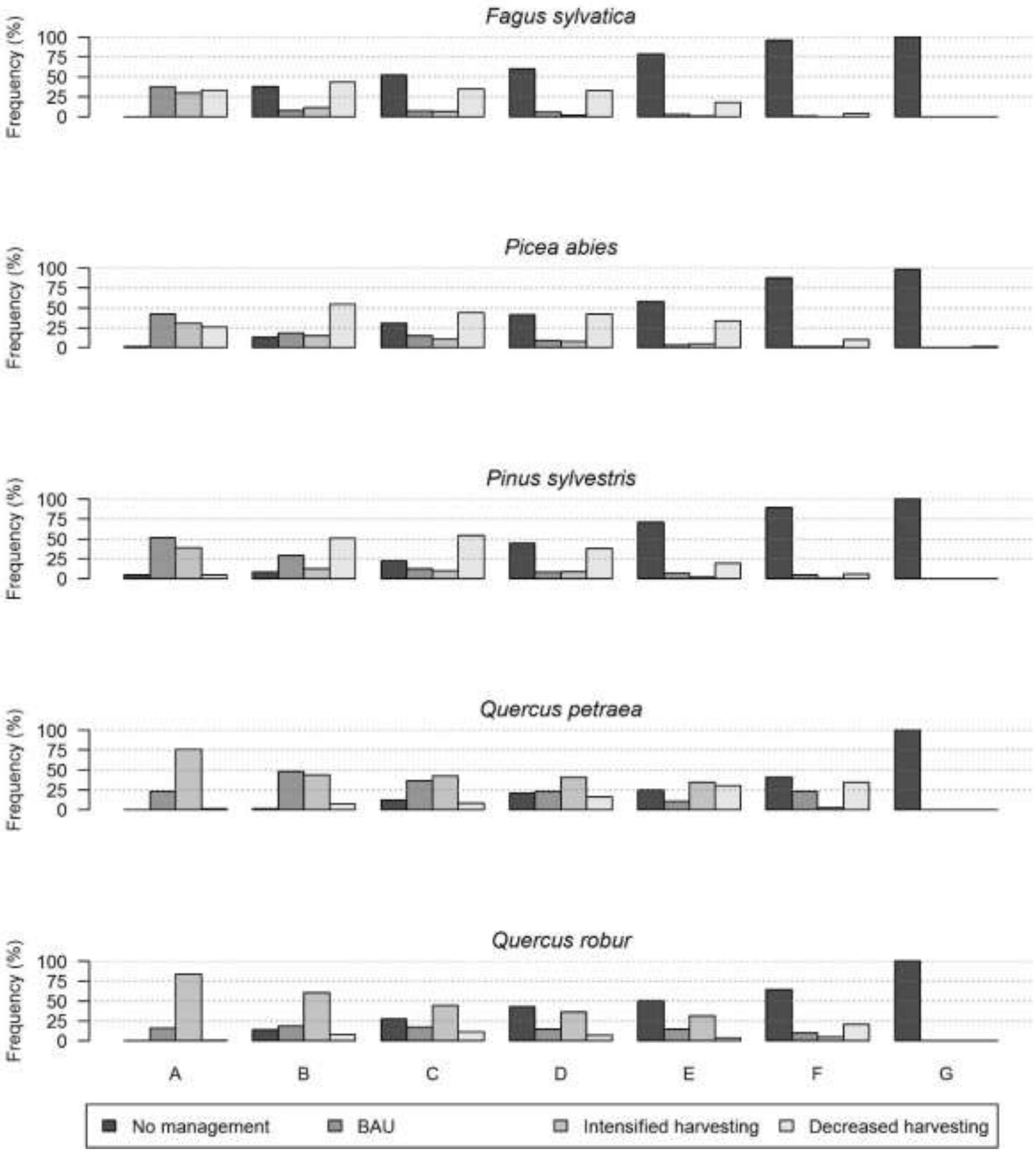

S3-Figure 6b. Relative frequency of each management intervention with increasing preference for carbon sequestration, considering a country-specific interest rate.

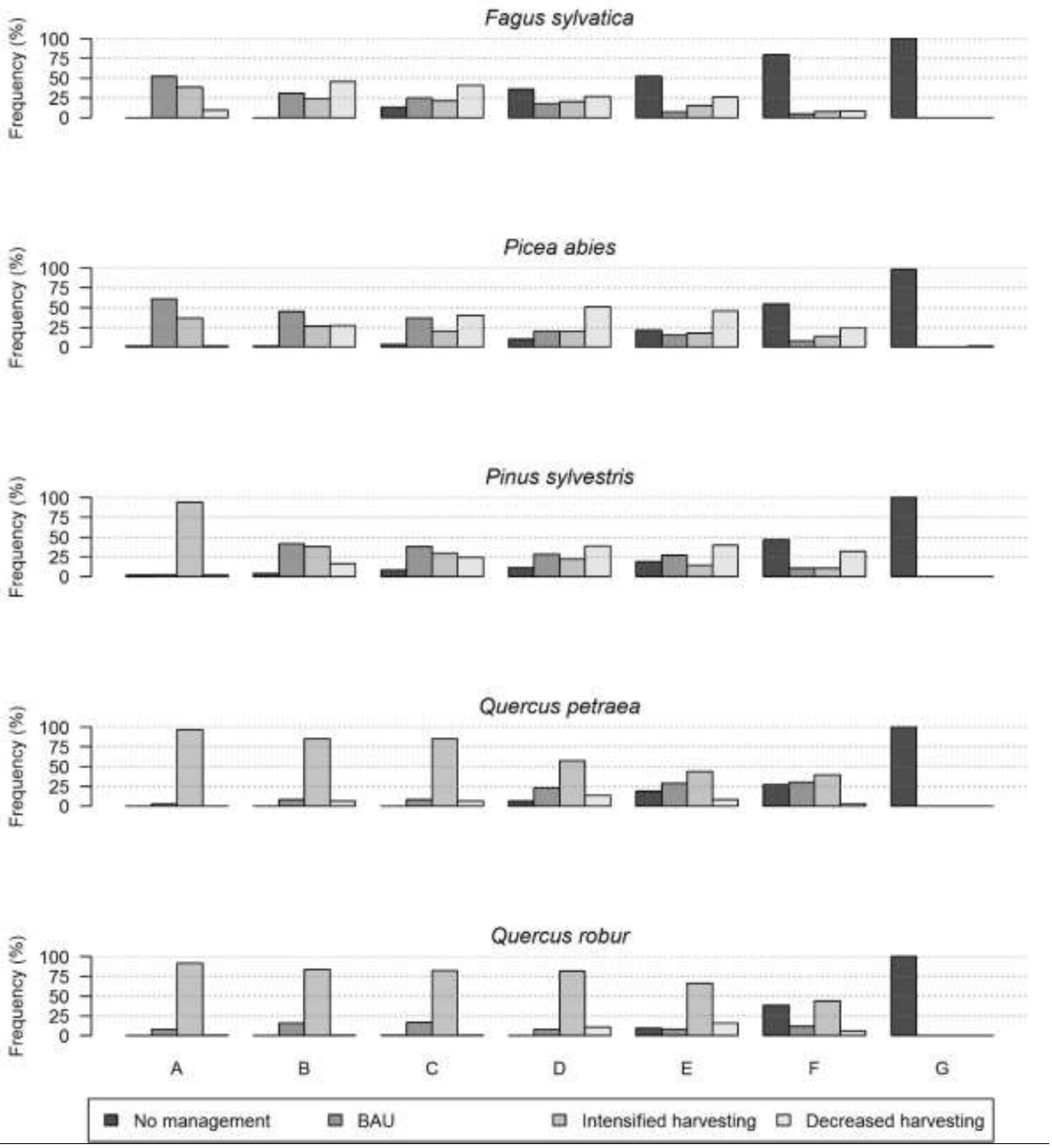

S3-Figure 6c. Relative frequency of each management intervention with increasing preference for carbon sequestration, considering a 2% interest rate.

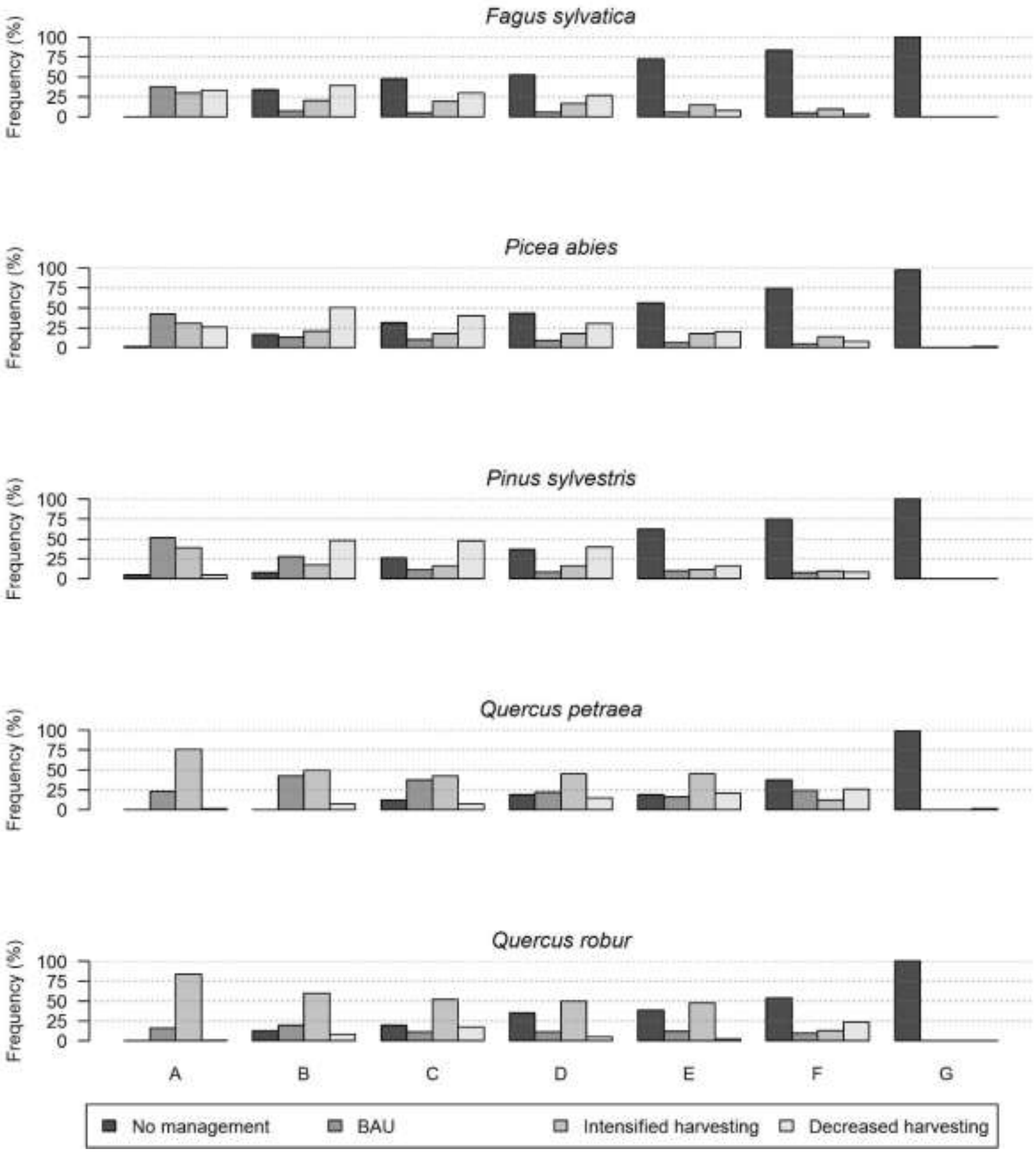

S3-Figure 6d. Relative frequency of each management intervention with increasing preference for carbon sequestration, considering a country-specific interest rate and no time preference for carbon sequestration.

Discussion on management interventions

Considering the results obtained in S3-Figure 5 we verified that a no management policy would maximize the carbon stored in forest structure. Some exceptions were observed for *Picea abies* and *Pinus sylvestris*, with a small proportion of other management interventions. These exceptions appeared in cases in which the forest stands suffered a high mortality rate with no management interventions, releasing the carbon stored. In such cases, management strategies with no intervention combined with one decreased wood harvesting intervention or BAU intervention were selected.

For balanced policies B to F, it was necessary to diversify management interventions. Taking into account the diverse behavior of different species in different environmental zones, it was necessary to adequately plan management actions according to forest responses in terms of NPV and carbon sequestration, applying country and species-specific management strategies. In general, decreased wood harvesting intensity appeared as the main course of action in order to increase carbon sequestration and maintaining suitable profitability levels for the conifer species and *Fagus sylvatica*. For the oak species, management strategies were mixed, likely as result of strong dissimilarities on forest development in different countries and the allocation of these areas to wood production due to the possibility of high profits. The interest rate altered the management intervention distribution for all species. For both oak species, the proportion of forest utilization increased for the 2% interest rate, compared to the 0% and country-specific interest rates. As interest rates were low for the countries in which oak species occurred, the management interventions were similar for the 0% and country-specific interest rates.

The increase in the interest rate from 0%to 2% implies a reduced contribution to the objective function given by the NPV along the simulation period, due to the increased discount factor. In this case, the lower contribution of the standing stocks and revenues occurring at later period of the simulation, it was more profitable to increase harvesting to benefit from early revenues, as growth rates became lower than the interest rate applied. Consequently, a forest conservation policy was adopted only with a very high preference for carbon sequestration. On the other hand, with the application of a 0% and country-specific interest rates (mostly below 1%), an increased contribution of the remaining stock at the end of the simulation period caused a decrease in harvesting intensity, even for management strategies maximizing NPV. Therefore, no thinning interventions were selected even with higher preference for NPV, e.g. under policy C.

When time preferences for carbon sequestration were disregarded, slightly higher wood utilization levels occurred (S3-Fig. 5d). The most significant differences were observed for policies E and F, with higher preference for carbon sequestration. When the discounting was applied (S3-Fig. 5b), the contribution of carbon sequestered earlier in the simulation period had a greater weight, thus it was necessary to reduce wood utilization starting earlier compared to the scenario with no time preference for carbon sequestration. With no urgency for carbon sequestration, management strategies with higher profitability, i.e. with more intense wood harvesting, still produced substantial levels of carbon sequestration allowing harvesting interventions even with higher preference for carbon sequestration.

References

1. Adams, D. M. et al. Minimum cost strategies for sequestering carbon in forests. *Land Economics* **75,** 360-374 (1999).
2. Anthoff, D. et al. Risk aversion, time preference, and the social cost of carbon. *Environmental Research Letters* **4,** 252 (2009).
3. Greenstone, M. et al. Estimating the social cost of carbon for use in us federal rulemakings: A summary and interpretation (National Bureau of Economic Research, 2011).
